# Ebola virus disease outbreak in Korea: use of a mathematical model and stochastic simulation to estimate risk

**DOI:** 10.4178/epih.e2019048

**Published:** 2019-11-24

**Authors:** Youngsuk Ko, Seok-Min Lee, Soyoung Kim, Moran Ki, Eunok Jung

**Affiliations:** 1 Department of Mathematics, Konkuk University, Seoul, Korea; 2 Department of Liberal Arts, Hongik University College of Engineering, Seoul, Korea; 3 Department of Cancer Control and Population Health, Graduate School of Cancer Science and Policy, National Cancer Center, Goyang, Korea

**Keywords:** Ebolavirus, Theoretical models, Disease outbreaks, Stochastic processes, Republic of Korea

## Abstract

**OBJECTIVES:**

According to the World Health Organization, there have been frequent reports of Ebola virus disease (EVD) since the 2014 EVD pandemic in West Africa. We aim to estimate the outbreak scale when an EVD infected person arrives in Korea.

**METHODS:**

Western Africa EVD epidemic mathematical model SEIJR or SEIJQR was modified to create a Korean EVD outbreak model. The expected number of EVD patients and outbreak duration were calculated by stochastic simulation under the scenarios of Best case, Diagnosis delay, and Case missing.

**RESULTS:**

The 2,000 trials of stochastic simulation for each scenario demonstrated the following results: The possible median number of patients is 2 and the estimated maximum number is 11 when the government intervention is proceeded immediately right after the first EVD case is confirmed. With a 6-day delay in diagnosis of the first case, the median number of patients becomes 7, and the maximum, 20. If the first case is missed and the government intervention is not activated until 2 cases of secondary infection occur, the median number of patients is estimated at 15, and the maximum, at 35.

**CONCLUSIONS:**

Timely and rigorous diagnosis is important to reduce the spreading scale of infection when a new communicable disease is inflowed into Korea. Moreover, it is imperative to strengthen the local surveillance system and diagnostic protocols to avoid missing cases of secondary infection.

## INTRODUCTION

Ebola virus disease (EVD), called hemorrhagic fever, is a systemic illness caused by the Ebola virus. EVD infection starts with symptoms such as fever, weakness, headache, myalgia, and pharyngitis (dry phase) after nine days latent period (range, 2 to 21 days), and then vomiting and diarrhea develop (wet phase). Internal and external hemorrhage can be found. The patient may recover, in 10–12 days from the disease onset [1-3]. EVD is usually transmitted through close contact within 1 m distance involving transfer of bodily fluid; in particular, substantial transmission occurs from dead patients [4,5].

The largest epidemic was in 2014–2015 in West Africa (Guinea, Liberia, and Sierra Leone) with a total of 28,616 infected patients and 11,310 casualties [6]. Since EVD is transmitted by close contact and have a high mortality, it is not likely to progress into a pandemic, but the location of first outbreak site (the border of 3 countries) and the Western African funeral rituals (embracing or kissing the deceased) made the spread much greater. Moreover, it was noted that the risk of infection amongst medical staff who had a relatively higher frequency of close contact with patients was substantially high [7-12]. In the 2014 epidemic, USA, Spain, and UK, which have dispatched medical teams, also experienced casualties and cases of infection [8-10]. In the USA, it was reported that 2 cases of patients were returned home in the latent stage of EVD. One of them was not a healthcare worker (HCW), and in a related event, 2 additional cases of infection among medical staff have occurred domestically in the USA [13-15].

The mathematical modeling of infectious diseases plays a crucial role to overcome the limitation of the induction method that uses experimental observation. Many Western Africa EVD mathematical models have been developed, and ones that take the funeral culture into account are also introduced [16,17].

By using the mathematical modeling and stochastic simulation, this study aims to analyze the importance of timing of diagnosis of the first case and early isolation of patients. The number of new patients is estimated in the event that a EVD infected patient arrives in Korea, assuming the delay in diagnosis for the first patient, and assuming the failure to detect EVD until several cases of secondary infected patients are found.

## MATERIALS AND METHODS

### Mathematical model

**Figure 1.**
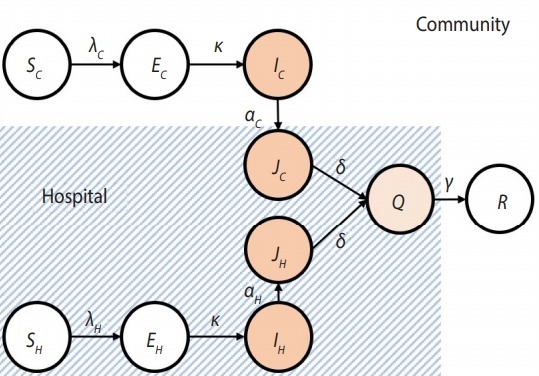
Flowchart of Ebola virus disease model. Light blue color was used to demarcate the areas of the hospitals from the local community. S, Susceptible; E, Exposed; I, Infectious; J, Hospitalized; Q, Isolation-treated; and R, Recovered groups, respectively. Please refer the methods section for the subscripts of each item.

One of the significant transmission routes of EVD is known to be the infection of medical staff who have frequent contact with patients, and the transmission by the infected staff. Therefore, this study divided the entire population into a HCW group and the Community (C) group to consider the heterogeneity of. After dividing the population into the HCW and C groups, each group was subdivided into the Susceptible (S), Exposed (E), Infectious (I), Hospitalized (J), Isolation-treated (Q), and Recovered (R) groups. During the isolated treatment period following the confirmed diagnosis, it is assumed that no spread of infection would occur. Since the J and R groups in the HCW and C groups have the same behavioral patterns, contact rates, and rates of transmission, they were not divided further into medical and non-medical groups. Figure 1 shows the flow of spread of EVD, and light blue color was used to demarcate the areas of the hospitals from the local community.

To establish the mathematical model of Korean EVD, the Western African EVD epidemic model (Supplementary Material 1) from the 2014 Sierra Leone data was modified to fit the circumstances of the Korean healthcare system. Firstly, unlike Western Africa, where infection from the funeral customs was an important route for spreading, it was assumed that the Korean healthcare environment would not lead to infection through contact with the bodies of the deceased. In addition, all patients with EVD symptoms were assumed to be hospitalized and isolated. The duration from the day of onset of EVD symptoms to the day of hospitalization and isolation periods was set with reference to cases imported into the USA.

The S group, which has never contracted Ebola virus, is exposed to the virus through infected and hospitalized patients. It has been hypothesized that the spread of virus would be different depending on whether the infected and hospitalized patients are local C members or HCWs.

The subscripts of each item, *H* and *C*, indicate the HCW group and C group, respectively. The model constant *β* specifies the rate of transmission of infection, and the subscripts onto *β* demonstrate the route of transmission. For instance, *β_CH_* refers to the rate of spread in the case where infected persons from the community spread the virus to HCWs. The *λ* was set by combining the different rates of infection from each group. Patients exposed to the virus become infected and can propagate the virus following a certain period. The constant κ is the rate of progression in the symptom onset of EVD, and 1/*κ* signifies the mean latent period of EVD. Patients with the onset of symptoms will be hospitalized after a certain period of time. The rate of hospitalization of infected patients is indicated by α, and 1/α is the average duration from onset of symptoms to hospitalization. Hospitalized patients become confirmed EVD cases through confirmatory diagnostic testing, and it is assumed that confirmed EVD patients will be isolated thereafter. The ratio of hospitalized patients to be isolated after confirmed diagnoses is defined as constant *δ*, and 1/*δ* is the average duration of hospitalized patients to be isolated after their confirmed diagnoses. The constant *γ* indicates the recovery rate of the isolated patients, and 1/*γ* signifies the mean isolation period for recovery. It is presumed that after the patients have been isolated, no further propagation can occur to susceptible patients. The mathematical model of the spread of EVD infection is as follows:

$$\frac{dS_{C}}{dt}=-\lambda_{C}S_{C},\;\;\;\;\;\;\;\;\;\;\;\;\;\frac{dS_{H}}{dt}=-\lambda_{H}S_{H},\;\frac{dE_{C}}{dt}=\lambda_{C}S_{C}-\kappa E_{C,\;\;\;\;\;\;\;}\frac{dE_{H}}{dt}=\lambda_{H}S_{H}-\kappa E_{H},\;\frac{dI_{C}}{dt}=\kappa E_{C}-\alpha_{C}I_{C},\;\;\;\;\;\;\;\;\frac{dI_{H}}{dt}=\kappa E_{H}-\alpha_{H}I_{H,}\;\frac{dJ_{C}}{dt}=\alpha_{C}I_{C}-\delta J_{C},\;\;\;\;\;\;\;\frac{dJ_{H}}{dt}=\alpha_{H}I_{H}-\delta J_{H,}\;\frac{dQ}{dt}=\delta \left(J_{C}+J_{H}\right)-\gamma Q,\;\frac{dR}{dt}=\gamma Q,\;\lambda_{C}=\frac{\beta_{CC}I_{C}+\beta_{HC}I_{H}+\beta_{JC}\left(J_{C}+J_{H}\right)}{N},\;\lambda_{H}=\frac{\beta_{CH}I_{C}+\beta_{HH}I_{H}+\beta_{JH}\left(J_{C}+J_{H}\right)}{N},\;\;\;\;\;\;\;\;\;\;N=S_{C}+E_{C}+I_{C}+J_{C}+S_{H}+E_{H}+I_{H}+J_{H}+Q+R.\;$$

The definition and values of the parameters in the spread model of Korean EVD are shown in Table 1. The rate of transmission of infection (*β*) is estimated by using the World Health Organization (WHO) report of new EVD case data for Sierra Leone at the time of the 2014 Western African EVD. EVD epidemic data were estimated by comparing data from the WHO’s weekly accumulating patient number and that of the model from corresponding dates. It was also based on rates of transmission of infection that minimize the squared difference between the number of new cases from the data and from the model estimation, using the least square fitting method [6]. In parameter estimation, the direction of spread between the C and HCW groups was not considered. In other words, *β_HC_*=*β_CH_*. The details for estimating the rate of EVD spread using the Sierra Leone data are described in the Supplementary Material 2, and all rates of propagation, except for spread by dead bodies, are identical to the Western African model. The total number of susceptible patients (*S_C_* [0]+*S_H_* [0]) in the model is 51,709,000, within which the number of HCWs (*S_H_* [0]) is 558,970 [18,19]. The first imported patient is assumed to be one from the latent patients in the community (*E_C_*[0]=1). The basic reproductive number of EVD infections (*R_0_*) is calculated using the Next-generation method [20].

Based on the mathematical model of the domestic spread of EVD transmission, estimates of possible number of patients and duration of outbreak for each corresponding response scenario for domestic imported cases of EVD were made with the Gillespie algorithm, which is a stochastic model simulation that is run by units of individual events [21].

Further detailed description of the mathematical modeling and stochastic simulation which are used in this research is provided in the supporting information.

### Ebola virus disease patient arrival scenario

We assume the outbreak situation that EVD infected C member in latent period, which is identical to one *E_C_* in the mathematical model, arrives in Korea. Later he or she experience symptoms onset and should be hospitalized, and following confirmation of EVD diagnosis, he or she should be isolated. EVD can be transmitted from patients who have experienced the onset of symptoms but are not isolated yet (I, J). It is assumed that the first confirmed diagnosis of an EVD patient, the intervention would commence. If the intervention policy is activated, it is presumed that the duration from symptom onset to hospitalization would decrease from 4 days to 2 days in the C and 3 days to 2 days in the hospital, and that the duration of confirmed diagnosis to isolation following hospitalization would be shortened from 2 days to 1 day. It is likewise expected that people would socially limit close contact, which would result in a 20% decrease of transmission rates (*β_CC_*, *β_HC_*, *β_HH_*, and *β_JC_*), and also that, within the hospital, the transmission rate to HCWs by patients (*β_JH_*) would decrease by 60% (Table 2).

Arrival of EVD patient and response scenarios are composed of the Best scenario (SI) and two additional scenarios as shown below.

SI: Following hospitalization of the first EVD patient, EVD diagnosis and isolation is immediately performed.

Diagnosis delay scenario (SII): Following hospitalization of the first EVD patient, there is a 3-day or 6-day delay in the diagnosis.

Case missing scenario (SIII): The EVD case is not recognized until the hospitalization of 1 or 2 secondary infected patients.

### Ethics statement

Ethical approval was not required because this study was based on a series of computer simulations and did not use any human or animal data in the study.

## RESULTS

To analyze the patterns of transmission of the EVD infection after a community member in a latent stage has been arrived, stochastic simulation results were obtained by 2,000 trials. In the analysis of simulation results, the total number of patients is the sum of and from the mathematical model.

**Figure 2.**
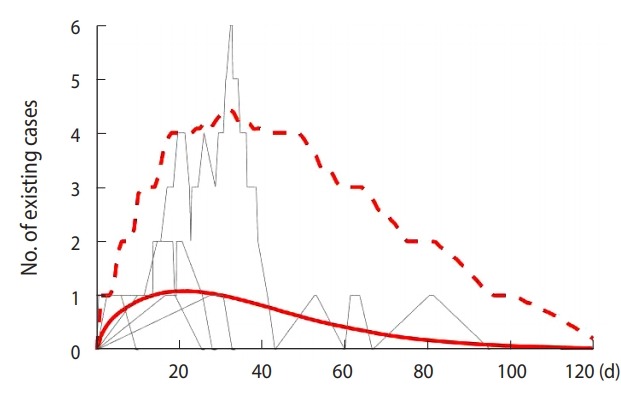
Estimated number of existing cases after the first patient of Ebola virus disease entered Korea, using model simulations. Grey curves are randomly sampled 5 simulations. Red curve indicates mean number of existing cases and dashed red curve indicates upper limit within 95% confidence interval.

Figure 2 depicts the changes of numbers of patients as the result from 2,000 trials in SI; more specifically, the number of patients of the 5 randomly selected results, mean of total number of patients from the entire simulation, and the upper bound of 95% confidence interval (CI) of patient number (a percentile of 97.5% of all 2,000 trials), are represented in gray curved lines, red solid lines, and red dashed lines, respectively. The results demonstrated that there can be at the most 3.5 patients within the 95% CI after 25 days the first imported case of a patient, and that the number of patients remains less than 1 on average.

**Figure 3.**
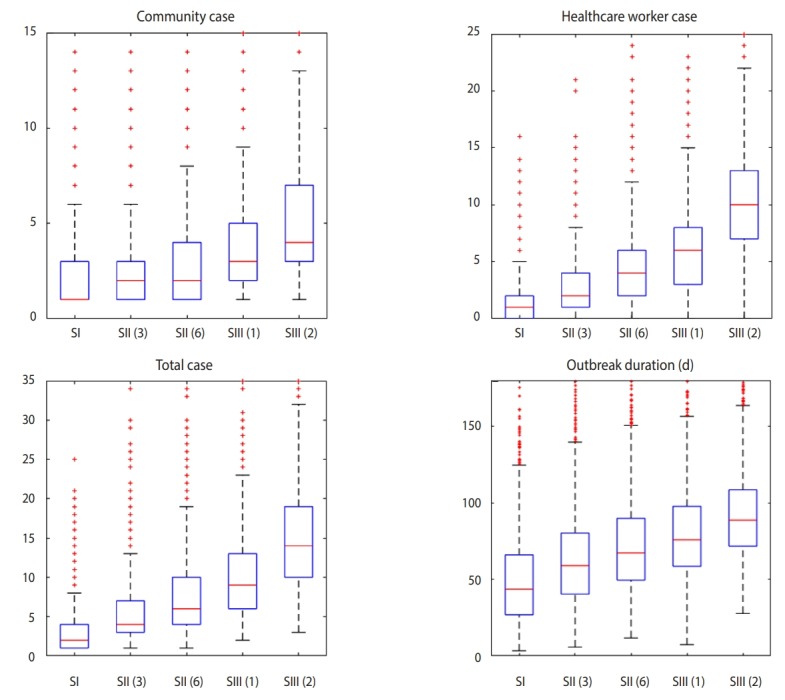
Boxplot results by scenario: SI, Best case; SII, Diagnosis delay (d); SIII, Case missing (n). On each box, red horizontal line indicates the median and bottom and top of each box indicates 25th and 75th percentiles, respectively. Whiskers reach extreme points within the confidence interval. Red crosses are outliers of the stochastic simulation.

Figure 3 shows the distribution of each simulation result as a box plot, regarding the predicted number of patients and epidemic duration that points to the recovery of the last patients. Table 3 lists the results for each EVD response scenario; total number of patients for C group and HCW group; median and maximum, and minimum values of the CI; maximum number of new cases per day; epidemic duration; and the probability of estimated total cases having greater than or equal to 10, 20, and 30 patients, which is calculated by the ratios of number of trials with those patients numbers, as well as the basic reproductive number before the intervention.

In SI, the median for the total predicted number of patients was calculated as 2 and the maximum number as 11. The probability of having more than or equal to 10 patients was 4.1%, and median epidemic duration was approximately 44 days. It is of interest to note that the results are similar to that of EVD cases in the USA [13-15].

In SII with 3-day of diagnosis delay, a total of 5 cases (2 in C and 3 in HCW) are predicted to occur. The maximum number of new patients in a day is predicted to be 2 at day 23 from the first domestic imported case, and the epidemic duration is estimated to be approximately 2 months (58 days). If there is a 6-day of diagnosis delay, a total of 7 patients are expected, and the duration of epidemics would extend to 69 days.

In SIII with 1 case missing, the median number of infected patients becomes 8 (3 in C and 5 in HCW). At the most 3 new patients would be observed per day in the day 24 after the first EVD patient arrives, and the epidemic duration is expected approximately 77 days. Impact of 1 case missing is equal nearly an 8-day diagnosis delay in SII, within around 88 days of epidemic, the median number of infected cases would involve 15 patients (5 in C and 10 in HCW), and it is predicted that at day 24, at the most 5 patients would appear per day. When there are 2 case missing, there is a 75.7% chance that more than 10 new cases would occur.

## DISCUSSION

A delay in the diagnosis of the first EVD patient arrival and secondary cases have resulted in an increase in the number of EVD patients and epidemic duration. If the assumption is made that no diagnosis is confirmed nor the first EVD patient is isolated for 3 days after he or she has been hospitalized (SII: 3 days), it was found that 1 new secondary case in the C and 2 cases from HCWs would additionally occur compared to the numbers from the prompt diagnosis and isolation against the first imported case (SI); The epidemic duration is supposed to increase by 14 days, and the probability of having more than 10 cases is 10.5%, which is 6.4% points higher than that of the prompt diagnosis and isolation scenario against the first domestic case. When there is 6 days of diagnosis delay, 5 more secondary infection cases (2 C and 3 HCW patients) are estimated to occur, and epidemic duration is likely to be 25 days longer, compared to those in the scenario involving early diagnosis and isolation of the first case. The probability of having more than 10 new patients is found to be 21.2%.

In SIII with 1 case missing, it is calculated that additional 2 and 4 new secondary cases in the C and HCWs would be involved compared to SI, respectively. The outbreak duration is increased by 33 days. The probability of involving more than 10 new patients is 32.8%, which is 8 times greater compared with SI, while the probability involving more than 20 patients is shown to be 1.6%. It is predicted that when there are 2 case missing, additional 13 cases with secondary infection would occur, resulting in a total of 15 patients. The results of 2 case missing have shown a much greater scale of epidemic; the probability of having more than 10 patients is more than doubled, and that of having more than 20 patients is 12.68 times higher compared with missing a case until 1 secondary infection patient is found. In particular, when compared to SI, the total number of patients increased from 2 to 15, and the probability of having more than 10.0% of the total predicted patients increased from 4.1% to 75.7%. The simulation results demonstrate that an EVD epidemic can arise when secondary infection occurs because of missed diagnosis of the primary infection case.

In the simulation, most of the increase in total patients in SII and SIII compared with that in SI is from the HCW group, as characteristics of EVD transmission that the risk of infection is shown to be higher for HCWs.

In this study, EVD mathematical model in Korea was built and simulations were performed that predict the scale of new patients and the duration of epidemics, considering the intervention policy similar to that in USA, under the SII and SIII. If EVD outbreak situation similar to the USA case is considered, it is expected that there would be 1 secondary infected HCW. However, if there is a 6-day of diagnosis delay, it was observed that a total of 7 new patients are expected. When there are 2 missing cases, the total number of patients becomes 15 in terms of median number, and within the CI, a maximum number of 35 cases can occur.

There has been no EVD outbreak in Korea, and thus, the parameters of the model were estimated from the Western Africa EVD epidemic. The transmission rate in Korea cannot be the same as that of Sierra Leone where social contact patterns are dissimilar. However, as was observed in 2015 Middle East respiratory syndrome epidemic, which demonstrated that Korea can have higher rate of spread than that of the country of the origin, it cannot be ascertained that the transmission rate would be low in Korea.

Simulation results showed that, it is highly critical that the first infected patient undergo confirmatory diagnosis as soon as possible, followed by promptly activating the intervention policy such as isolation. Therefore, to facilitate prompt identification of patients and to diagnosis, it can be emphasized that it is necessary to construct a contingency system such as monitoring and tracing for infectious diseases, as well as identification of international travel history.

## Figures and Tables

**Table 1.** Model parameters to estimate outbreak size of Ebola virus disease

Symbol	Description	Value	Reference
*β_CC_*	Transmission rate between community members	0.1352	Data-fitting
*β_HC_, β_CH_*	Transmission rate between community members and HCWs	0.8110	Data-fitting
*β_HH_*	Transmission rate between HCWs	0.8110	Data-fitting
*β_JC_, β_CJ_*	Transmission rate between hospitalized patients and community members	0.0405	Data-fitting
*β_JH_*	Transmission rate between hospitalized patients and HCWs	45.5512	Data-fitting
1/*κ*	Incubation period	11 d	[1]
1/*α_C_*	Period of symptom onset to hospitalization of community members	4 d	[13-15]
1/*α_H_*	Period of symptom onset to hospitalization of HCWs	3 d	[13-15]
1/*δ*	Period of hospitalization to isolation	2 d	[13-15]
1/*γ*	Period of isolation to recovery	14 d	[13-15]

HCWs, healthcare workers.

**Table 2.** Model parameters before and after intervention for Ebola virus disease outbreak

Symbol	Pre-behavior change	Post-behavior change
*β_CC_*	0.1352	0.1082
*β_HC_*	0.0811	0.0649
*β_HH_*	0.0811	0.0649
*β_JC_*	0.0405	0.0324
*β_JH_*	45.5512	18.2205
1/*α_C_*	4 d	2 d
1/*α_H_*	3 d	2 d
1/*δ*	2 d	1 d

**Table 3.** Estimated number of cases and duration of outbreak of Ebola virus disease by scenario

	Best case	Diagnosis delay (d)		Case missing (n)	
		3	5	1	2
Estimated case, median (Min-Max, n)					
Total	2 (1-11)	5 (1-14)	7 (1-20)	8 (1-24)	15 (1-35)
Community	1 (1-6)	2 (1-6)	3 (1-8)	3 (1-9)	5 (1-13)
Healthcare worker	1 (0-5)	3 (0-8)	4 (0-12)	5 (0-15)	10 (0-22)
Peak size of existing case (Max, n)	1 (3)	2 (4)	2 (6)	3 (7)	5 (10)
Outbreak duration, median (Min-Max, d)	44 (3-121)	58 (5-139)	69 (11-152)	77 (7-156)	88 (27-164)
Probability of estimated total case (%)					
30 or more	0.0	0.0	0.3	0.0	3.2
20 or more	0.3	1.1	3.0	1.6	20.3
10 or more	4.1	10.5	21.2	32.8	75.7
Reproductive (pre-behavior change, n)	2.42	4.05	6.05	7.74	10.49

Min, minimum; Max, maximum.

## SUPPLEMENTARY MATERIALS

Korean version is available at http://www.e-epih.org/.
