# Supplementary material for: Ebola virus disease outbreak in Korea: use of a mathematical model and stochastic simulation to estimate risk
Source: Epidemiol Health. 2019 Nov 24;41:e2019048. doi: 10.4178/epih.e2019048 (PMC7005456; doi:10.4178/epih.e2019048)
Supplement: Supplementary file 1 [file epih-41-e2019048-supplementary.pdf]

Supporting information

에볼라바이러스병의 국내 확산 위험도 예측: 수학적 모델링과 확률 시뮬레이션을 통하여

Risk estimation of Ebola virus disease outbreak in the Republic of Korea: using mathematical model and stochastic simulation

1. 2014 년 서아프리카 EVD 유행의 수리모델

의료진과 지역사회 환자의 감염이질성을 고려하기 위하여 전체 인구를 의료종사자(healthcare worker; HCW) 집단과 일반 지역사회(community; C) 집단으로 나누었다. 의료종사자와 일반 지역사회 인구를 각각 감수성자(Susceptible; S), 잠복기 환자(Exposed; E), 감염자(Infectious; I), 내원환자(Hospitalized; J), 회복자(Recovered; R), 그리고, 사망자(Funeral; F) 그룹으로 구분하였다. Figure 1 은 EVD 전파의 감염흐름도를 나타낸 것이다.

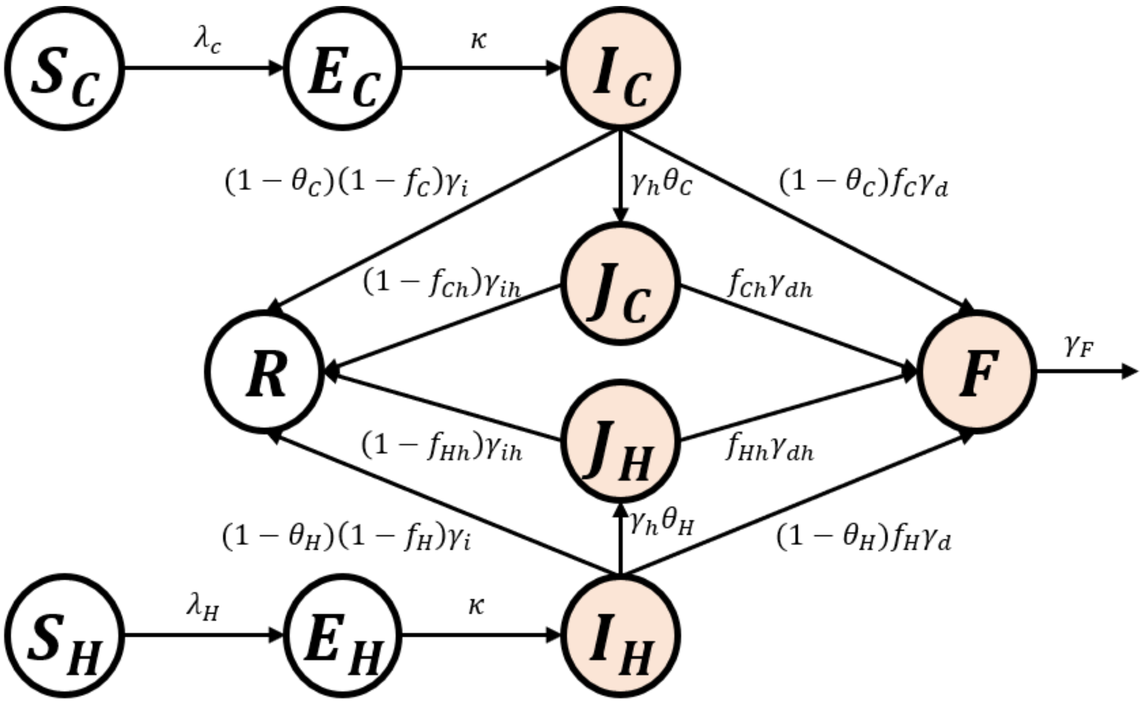

Figure 1 Flowchart of EVD model of 2014 Wester Africa EVD epidemic

β는 각 감염 전파율이며 아래첨자는 감염원과 피감염원을 순서대로 나타낸다. 감염이 된 환자는 잠복기를 거친 이후 증상이 나타난 감염자가 된다. 감염자는 입원하지 않고 회복(γ_i)하거나 사망(f)할 수 있으며, 입원(γ_h)을 한 뒤에도 회복(γ_{ih})하거나 사망(γ_{dh})할 수 있다. 지역사회 집단과 의료종사자 집단의 회복률과 사망률, 입원률은 모두 다르므로 아래첨자를 이용하여 구분하였다. 장례문화의 전파를 고려하여 사망을 한 사람은 장례절차(γ_F)를 거치는 동안 감염을 전파시킨다. WHO 의 중재를 고려하기 위하여 특정 시점(τ) 이후로 전파율(β)이 감소하며 환자에 의한 전파율($\beta_{CC}, \beta_{HC}, \beta_{HH}, \beta_{HC}, \beta_{JC}, \beta_{JH}$)과 사망자에 의한 전파율($\beta_{FC}, \beta_{FH}$)이 각자 다른 비율로 지수적으로 감소한다 가정하였다 [1, 2, 3]. 수리모델은 다음과 같다.

$$\frac{dS_C}{dt} = -\frac{\left(\beta_{CC}I_C + \beta_{HC}I_H + \beta_{JC}(J_C + J_H) + \beta_{FC}F\right)}{N}S_C,$$

$$\frac{dE_C}{dt} = \frac{(\beta_{CC}I_C + \beta_{HC}I_H + \beta_{JC}(J_C + J_H) + \beta_{FC}F)}{N}S_C - \kappa E_C,$$

$$\frac{dI_C}{dt} = \kappa E_C - \left((1-\theta_C)(1-f_C)\gamma_i + (1-\theta_C)f_C\gamma_d + \gamma_{Ch}\theta_C\right)I_C,$$

$$\frac{dJ_C}{dt} = \gamma_{Ch}I_C - \left((1-f_{Ch})\gamma_{ih} + f_{Ch}\gamma_{dh}\right)J_C,$$

$$\frac{dS_H}{dt} = \lambda_H - \frac{\left(\beta_{CH}I_C + \beta_{HH}I_H + \beta_{JH}(J_C + J_H) + \beta_{FH}F\right)}{N}S_H,$$

$$\frac{dE_H}{dt} = \frac{\left(\beta_{CH}I_C + \beta_{HH}I_H + \beta_{JH}(J_C + J_H) + \beta_{FH}F\right)}{N}S_H - \kappa E_H,$$

$$\frac{dI_H}{dt} = \kappa E_H - \left((1-\theta_H)(1-f_H)\gamma_i + (1-\theta_H)f_H\gamma_d + \gamma_{Hh}\theta_H\right)I_H,$$

$$\frac{dJ_H}{dt} = \gamma_{Hh}\theta_H I_H - \left((1-f_{Hh})\gamma_{ih} + f_{Hh}\gamma_{dh}\right)J_H,$$

$$\frac{dR}{dt} = (1-\theta_C)(1-f_C)\gamma_i I_C + (1-f_{Ch})\gamma_{ih}J_C + (1-\theta_H)(1-f_H)\gamma_i I_H + (1-f_{Hh})\gamma_{ih}J_H,$$

$$\frac{dF}{dt} = (1-\theta_C)f_C\gamma_d I_C + f_{Ch}\gamma_{cdh}J_C + (1-\theta_H)f_H\gamma_d I_H + f_{Hh}\gamma_{dh}J_H - \gamma_F F.$$

의료종사자와 비의료종사자의 개인위생수준의 차이, 병원과 지역사회의 위생환경 차이로 인한 EVD 의 감염 전파율 차이를 고려하기 위해 전파율 감소 모수 p_1, p_2 를 고려하였다 ($0 < p_1, p_2 < 1$). 감염 전파 감소는 다음과 같이 가정하였다. 첫째, 의료종사자의 경우 EVD 전파 예방 교육(p_1)으로 인해 병원 밖에서도 EVD 전파율이 낮다. 둘째, 의료종사자가 비의료종사자에게 EVD 를 전파하는 전파율은 비의료종사자가 의료종사자에게 EVD 를 전파하는 전파과 같다고 가정한다. 셋째, 비의료종사자의 병원내 전파율은 지역사회에서의 전파율보다 낮은 전파율(p_2)을 가진다. 교육효과로 감염전파가 100% 차단되면 $p_{1,2} = 0$, 교육효과가 전혀 없다면 $p_{1,2} = 1$ 로 정의하였다.

모델의 전파율들은 다음과 같은 관계식으로 표현이 가능하다.

$$\beta_{CC} = \beta_I,$$

$$\beta_{CH} = \beta_{HC} = p_1\beta_I,$$

$$\beta_{JC} = p_2\beta_I,$$

$$\beta_{JH} = \beta_J,$$

$$\beta_{FC} = \beta_F,$$

$$\beta_{FH} = p_1\beta_F.$$

본 가정에 사용된 전파율 감소 모수 p_1, p_2 는 PRCC 방법을 통해 모델에 미치는 민감도를 분석하였다.

2. 2014 년 시에라리온 데이터를 이용한 EVD 전파율 추정

데이터는 2014 년 서아프리카 EVD 유행 중 시에라리온의 데이터를 이용하였으며, MATLAB 의 lsqcurvefit 을 이용하여 시에라리온 EVD 유행의 주간 누적 환자수 데이터와 모델에서의 해당 날짜에서의 누적 환자수 결과의 차를 최소화하는 방식으로 추정하였다. 추정된 모수들은 Table 1 에 표기되어 있으며 모델로부터 추정된 누적 환자수 곡선과 실제 데이터는 Figure 2 에 도식되었다.

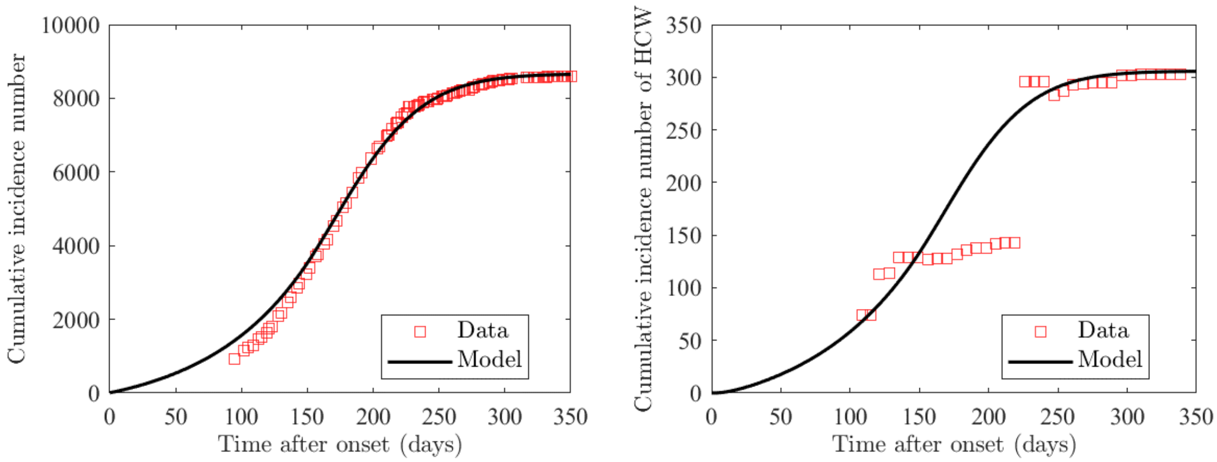

Figure 2 Parameter estimation results. The red squares indicate the cumulative number of EVD incidence during the 2014 EVD epidemic in Sierra Leone and black solid curve indicate fitted curve. Left graph and right graph display data-fitted results of community and HCW cases, respectively. Day 0 indicates the time when the first death case reported, May 26 2014, and day 350 is the time when the last case reported, May 11 2015.

Table 1 2014 Western Africa EVD epidemic model parameters

Symbol	Description	Value	Reference
β_I	Transmission rate of infectious individuals	0.1352	Data-fitting
β_J	Transmission rate of hospitalized to HCW	45.5512	Data-fitting
β_F	Transmission rate of funeral	0.1434	Data-fitting
p_1	Transmission reduction rate by HCW education	0.6	Assumed
p_2	Transmission reduction rate by hospitalization	0.3	Assumed
k_I	Transmission reduction rate by WHO intervention	0.0126	Data-fitting
k_F	Transmission reduction rate by WHO safe burial intervention	0.0010	Data-fitting
τ	Behavior change timing after outbreak	150	[2]
$1/\kappa$	Incubation period	11 days	[4]
$1/\gamma_h$	Period from onset to hospitalization	4 days	[5, 6]
$1/\gamma_i$	Period from onset to recovered	10 days	[5, 6]
$1/\gamma_d$	Period from onset to death	9.6 days	[5, 6]
$1/\gamma_{ih}$	Period from hospitalization to recovered	5 days	[5, 6]
$1/\gamma_{dh}$	Period from hospitalization to death	5 days	[5, 6]
$1/\gamma_F$	Period from death to burial	4 days	[7]
θ_C	Probability of community to be hospitalized	0.62	[8]
θ_H	Probability of HCW to be hospitalized	0.69	[8]
f_C	Fatality rate of not hospitalized community	0.84	[8]
f_H	Fatality rate of not hospitalized HCW	0.69	[8]
f_{Ch}	Fatality rate of hospitalized community	0.57	[8]
f_{Hh}	Fatality rate of hospitalized HCW	0.66	[8]
$S_C(0)$	Number of susceptible community	7079000	[9]
$S_H(0)$	Number of susceptible HCW	2000	[10]

모델과 데이터의 평균 제곱근 오차(Root mean square error)는 비의료종사자에 대해선 130.3058, 의료종사자에 대해선 45.4274 로 계산되었다. 시에라리온 EVD 유행 데이터 중 의료종사자 감염환자 데이터는 day 200 근처에서 급격히 늘며 누계 데이터임에도 감소하는 부분 또한 존재한다. 현재 단순한 형태의 모델에서는 이러한 급격한 변화를 표현할 수 없었다. 이는 당시 미보고 환자의 보고 혹은 데이터 집계 등에서 생길 수 있는 데이터의 불확실성일 수도 있고 급격한 환자 발생일 수도 있는 것으로 추측된다.

3. 전파율 감소 모수($\boldsymbol{p_1}$, $\boldsymbol{p_2}$)의 민감도 분석

전파율의 단순화를 위해 가정한 교육 및 입원에 의한 전파 변화율(p_1, p_2)에 대한 민감도 분석 실시하기 위하여 목적함수를 총 발생 환자수로 설정하고 편순위상관계수방법(partial rank correlation coefficient, PRCC)를 이용하여 모수 변화에 의한 민감도 분석을 진행하였다 [11]. 분석 결과는 Table 2 에 기술되었으며 두 전파 감소율 모두 비슷한 값으로 유의미한 수치를 나타내며 교육 및 입원에 의한 효과가 적을수록, 즉 p_1, p_2 값이 클수록, 총 발생 환자수는 증가한다고 해석할 수 있다.

Table 2 PRCC of parameters and p-value

Parameter	PRCC	p-value
p_1	0.2647	$<10^{-3}$
p_2	0.2802	$<10^{-3}$

References

[1] Francesconi P et al. Ebola hemorrhagic fever transmission and risk factors of contacts, Uganda. Emerging Infectious Diseases. 2003;9:1430–1437.

[2] Centers for Disease Control and Prevention. Overview, Control Strategies, and Lessons Learned in the CDC Response to the 2014–2016 Ebola Epidemic. Available from: https://www.cdc.gov/mmwr/volumes/65/su/su6503a2.htm

[3] Amanda Tiffany, Benjamin D. Dalziel, Hilary Kagume Njenge, Ginger Johnson, Roselyn Nugba Ballah, Daniel James, et al. Estimating the number of secondary Ebola cases resulting from an unsafe burial and risk factors for transmission during the West Africa Ebola epidemic. PLOS Neglected Tropical Diseases. 2017;11(6): e0005491. https://doi.org/10.1371/journal.pntd.0005491.

[4] World Health Organization. Ebola virus disease 2018. Available from: https://www.who.int/news-room/fact-sheets/detail/ebola-virus-disease

[5] Rivers CM, Logfren ET, Marathe M, Eubank S, Lewis BL. Modeling the impact of interventions on an epidemic of Ebola in Sierra Leone and Liberia, PLOS Currents Outbreaks. 2014 Nov 6. Edition 2. doi: 10.1371/currents.outbreaks.4d41fe5d6c05e9df30ddce33c66d084c.

[6] WHO Ebola Response Team. Ebola virus disease in West Africa - the first 9 months of the epidemic and forward projections. N. Engl. J. Med. 371(16), 1481–1495 (2014). Doi:10.1056/NEJMoa1411100; 21

[7] Folashade B Agusto, Miranda I Teboh-Ewungkem, Abba B Gumel. BMC Medicine 2015;13:96 https://doi.org/10.1186/s12916-015-0318-3.

[8] Health worker Ebola infections in Guinea, Liberia and Sierra Leone. World Health Organization; 21 May 2015.

[9] Worldometers. population statistics. Available from: https://www.worldometers.info/world-population/sierra-leone-population/

[10] Government of Sierra Leone, Ministry of Health and Sanitation. Annual Health Sector Performance Report 2016., 2016, 25-27.

[11] Marino S, Hogue IB, Ray CJ, Kirschner DE. A methodology for performing global uncertainty and sensitivity analysis in systems biology. J Theor Biol. 2008;254(1):178–196. doi:10.1016/j.jtbi.2008.04.011
