# Supplementary material for: Ebola virus disease outbreak in Korea: use of a mathematical model and stochastic simulation to estimate risk
Source: Epidemiol Health. 2019 Nov 24;41:e2019048. doi: 10.4178/epih.e2019048 (PMC7005456; doi:10.4178/epih.e2019048)
Supplement: Supplementary file 2 [file epih-41-e2019048-supplementary1.pdf]

Supporting information 2

Ebola virus disease outbreak in the Republic of Korea: use of a mathematical model and stochastic simulation to estimate risk

Data

To perform the parameter estimation of the model, data from the first case of mortality, on May 25, 2014, until the last reported case, May 11, 2015, have been utilized from the WHO's patient report data of the 2014 West Africa Sierra Leone EVD [2, 3]. The total population data of Sierra Leone were obtained from the population records of the 2014 Worldometers, and the corresponding datasets used were obtained from the estimated sums of the CHO/CHA, Env/public health, nursing, midwife, other allied health, medical officer, and nurse specialist data of the Sierra Leone government to apply similar criteria to the Korean healthcare worker data [12, 13].

The records for community population and healthcare professionals in Korea were obtained from the total figure of healthcare workers (doctors and nurses) per 10,000 people in the annals of the Korean Statistics Bureau and Ministry of Health and Welfare, respectively [7, 8].

Mathematical model

One of the significant transmission routes of EVD is known to be the infection of medical staff who have frequent contact with patients, and the transmission by the infected staff. Therefore, this study divided the entire population into a healthcare worker (HCW) group and the community (C) group to consider the heterogeneity of. After dividing the population into the HCW and C groups, each group was subdivided into the Susceptible (S), Exposed (E), Infectious (I), Hospitalized (J), Isolation-treated (Q), and Recovered (R) groups. During the isolated treatment period following the confirmed diagnosis, it is assumed that no spread of infection would occur. Since the J and R groups in the HCW and C groups have the same behavioral patterns, contact rates, and rates of transmission, they were not divided further into medical and non-medical groups. Figure 1 shows the flow of spread of EVD, and light blue color was used to demarcate the areas of the hospitals from the local community.

To establish the mathematical model of Korean EVD, the Western African EVD epidemic model (refer to supplemental data) from the 2014 Sierra Leone data was modified to fit the circumstances of the Korean healthcare system. Firstly, unlike Western Africa, where infection from the funeral customs was an important route for spreading, it was assumed that the Korean healthcare environment would not lead to infection through contact with the bodies of the deceased. In addition, all patients with EVD symptoms were assumed to be hospitalized and isolated. The duration from the day of onset of EVD symptoms to the day of hospitalization and isolation periods was set with reference to cases imported into the USA.

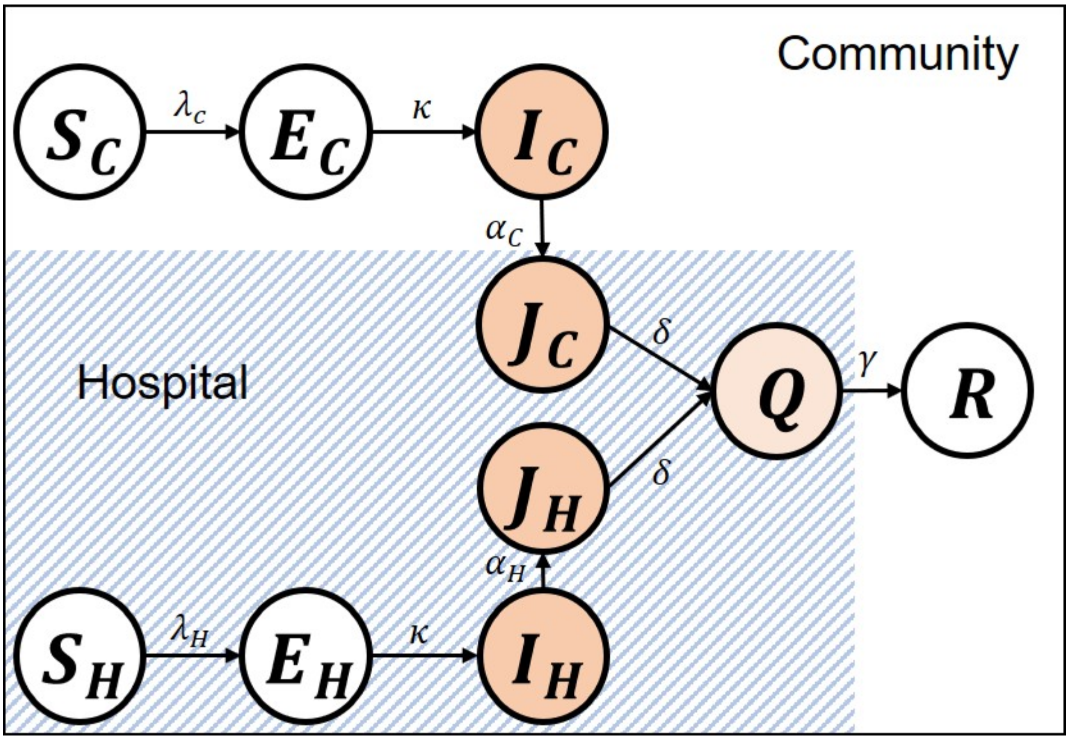

Figure 1 Flowchart of EVD model

The S group, which has never contracted Ebola virus, is exposed to the virus through infected and hospitalized patients. It has been hypothesized that the spread of virus would be different depending on whether the infected and hospitalized patients are local community members or healthcare workers. The subscripts of each item, H and C, indicate the healthcare worker group and community group, respectively. The model constant β specifies the rate of transmission of infection, and the subscripts onto β demonstrate the route of transmission. For instance, β_{CH} refers to the rate of spread in the case where infected persons from the community spread the virus to healthcare workers. The λ was set by combining the different rates of infection from each group. Patients exposed to the virus become infected and can propagate the virus following a certain period. The constant κ is the rate of progression in the symptom onset of EVD, and $1/\kappa$ signifies the mean latent period of EVD. Patients with the onset of symptoms will be hospitalized after a certain period of time. The rate of hospitalization of infected patients is indicated by α, and $1/\alpha$ is the average duration from onset of symptoms to hospitalization. Hospitalized patients become confirmed EVD cases through confirmatory diagnostic testing, and it is assumed that confirmed EVD patients will be isolated thereafter. The ratio of hospitalized patients to be isolated after confirmed diagnoses is defined as constant δ, and $1/\delta$ is the average duration of hospitalized patients to be isolated after their confirmed diagnoses. The constant γ indicates the recovery rate of the isolated patients, and $1/\gamma$ signifies the mean isolation period for recovery. It is presumed that after the patients have been isolated, no further propagation can occur to susceptible patients. The mathematical model of the spread of EVD infection is as follows:

$$\frac{dS_C}{dt} = -\lambda_C S_C, \qquad \frac{dS_H}{dt} = -\lambda_H S_H,$$

$$\frac{dE_C}{dt} = \lambda_C S_C - \kappa E_C, \qquad \frac{dE_H}{dt} = \lambda_H S_H - \kappa E_H$$

$$\frac{dI_C}{dt} = \kappa E_C - \alpha_C I_C, \qquad \frac{dI_H}{dt} = \kappa E_H - \alpha_H I_H,$$

$$\frac{dJ_C}{dt} = \alpha_C I_C - \delta J_C, \qquad \frac{dJ_H}{dt} = \alpha_H I_H - \delta J_H,$$

$$\frac{dQ}{dt} = \delta(J_C + J_H) - \gamma Q,$$

$$\frac{dR}{dt} = \gamma Q,$$

$$\lambda_C = \frac{\beta_{CC} I_C + \beta_{HC} I_H + \beta_{JC}(J_C + J_H)}{N},$$

$$\lambda_H = \frac{\beta_{CH} I_C + \beta_{HH} I_H + \beta_{JH}(J_C + J_H)}{N},$$

$$N = S_C + E_C + I_C + J_C + S_H + E_H + I_H + J_H + Q + R.$$

The definition and values of the parameters in the spread model of Korean EVD are shown in Table

1. The rate of transmission of infection (β) is estimated by using the WHO report of new EVD case data for Sierra Leone at the time of the 2014 Western African EVD. EVD epidemic data were estimated by comparing data from the WHO's weekly accumulating patient number and that of the model from corresponding dates. It was also based on rates of transmission of infection that minimize the squared difference between the number of new cases from the data and from the model estimation, using the least square fitting method [2]. In parameter estimation, the direction of spread between the community and healthcare worker groups was not considered. In other words, $\beta_{HC} = \beta_{CH}$. The details for estimating the rate of EVD spread using the Sierra Leone data are described in the supplementary data, and all rates of propagation, except for spread by dead bodies, are identical to the Western African model. The total number of susceptible patients ($S_C(0)+S_H(0)$) in the model is 51,709,000, within which the number of healthcare workers ($S_H(0)$) is 558,970 [7, 8]. The first imported patient is assumed to be one from the latent patients in the community ($E_C(0) = 1$).

Table 1: Model parameters to estimate outbreak size of Ebola Virus Disease

Symbol	Description	Value	Reference
β_{CC}	Transmission rate between community members	0.1352	Data-fitting
β_{HC}, β_{CH}	Transmission rate between community members and HCWs	0.811	Data-fitting
β_{HH}	Transmission rate between HCWs	0.811	Data-fitting
β_{JC}, β_{CJ}	Transmission rate between hospitalized patients and community members	0.0405	Data-fitting
β_{JH}	Transmission rate between hospitalized patients and HCWs	45.5512	Data-fitting
$1/\kappa$	Incubation period	11 days	[1]
$1/\alpha_C$	Period of symptom onset to hospitalization of community members	4 days	[4-6]
$1/\alpha_H$	Period of symptom onset to hospitalization of HCWs	3 days	[4-6]
$1/\delta$	Period of hospitalization to isolation	2 days	[4-6]
$1/\gamma$	Period of isolation to recovery	14 days	[4-6]

In the mathematical model of EVD infection transmission, the basic reproductive number of EVD infections (R_0) calculated by the Next-generation method is as follows [9]. Here, N_C and N_H refer to the community and healthcare worker population, respectively.

$$R_0 = \frac{A + \sqrt{A^2 + 4B}}{2},$$

$$A = \left(\frac{\beta_{CC}}{\alpha_C} + \frac{\beta_{JC}}{\delta}\right) p_C + \left(\frac{\beta_{HH}}{\alpha_H} + \frac{\beta_{JH}}{\delta}\right) p_H$$

$$\mathrm{B} = p_C p_H \left(\frac{\beta_{HC}\beta_{JC}}{\alpha_C \delta} + \frac{\beta_{HC}\beta_{JH}}{\alpha_H \delta} + \frac{\beta_{HC}^2}{\alpha_C \alpha_H} - \frac{\beta_{CC}\beta_{HH}}{\alpha_C \alpha_H} - \frac{\beta_{CC}\beta_{JH}}{\alpha_C \delta} - \frac{\beta_{HH}\beta_{JC}}{\alpha_H \delta}\right),$$

$$p_C = \frac{N_C}{N}, \qquad p_H = \frac{N_H}{N}.$$

The basic reproductive number of infection (R_0), when calculated based on 𝕊I, was 2.42 before the activation of interim policy, which decreases to 0.76 following the policy. In case of delay in diagnosis scenario (𝕊II), a delay of 3 days (6 days) causes the first case patient to show the basic reproductive number of 4.05 (6.05) which is higher than the previous value of 2.42. Besides, if the duration between confirmation of diagnosis and isolation is shortened from the previous mean of 2 days to 6 hours, it was shown that the basic reproductive number is estimated to be 0.99, which is less than 1, even without activation of the interim policy.

Based on the mathematical model of the domestic spread of EVD transmission, estimates of possible number of patients and duration of outbreak for each corresponding response scenario for domestic imported cases of EVD were made with the Gillespie algorithm, which is a stochastic model simulation that is run by units of individual events [10]. In this study, outbreak duration is defined as the time from arrival of the first patient to recovery of the last patient. In the stochastic simulation by the Gillespie algorithm, the duration between events is inversely related to the sum of propensity related to infection, hospitalization, isolation, and recovery. The propensity of each event is listed in Table 2.

Table 2: Possible transitions and propensities in the model

Transitions	Propensity
$S_C \to S_C - 1,\ E_C \to E_C + 1$	$\frac{\beta_{CC} I_C + \beta_{HC} I_H + \beta_{JC}(J_C + J_H)}{N} S_C$
$E_C \to E_C - 1,\ I_C \to I_C + 1$	κE_C
$I_C \to I_C - 1,\ J_C \to J_C + 1$	$\alpha_C \mathrm{I}_C$
$J_C \to J_C - 1,\ Q \to Q + 1$	$\delta \mathrm{J}_C$
$S_H \to S_H - 1,\ E_H \to E_H + 1$	$\frac{\beta_{CH} I_C + \beta_{HH} I_H + \beta_{JH}(J_C + J_H)}{N} S_H$
$E_H \to E_H - 1,\ I_H \to I_H + 1$	κE_H
$I_H \to I_H - 1,\ J_H \to J_H + 1$	$\alpha_H \mathrm{I}_H$
$J_H \to J_H - 1,\ Q \to Q + 1$	$\delta \mathrm{J}_H$
$Q \to Q - 1,\ \mathrm{R} \to \mathrm{R} + 1$	γQ

Sensitivity analysis

Using the partial rank correlation coefficient (PRCC), we have analyzed the effects of changes in parameters in the mathematical model on model outcome [11]. In this study, the rate of transmission of infection (β_{CC}, β_{HC}, β_{HH}, β_{JC}), duration of symptom onset to hospitalization ($1/\alpha_C$, $1/\alpha_H$), and duration from confirmed diagnosis of hospitalized patient to isolation ($1/\delta$) were set as variables, and subsequently, the number of secondary infection cases during the transmission period were set as a function of the corresponding variable. In the analysis of susceptibility, the cases of delay in diagnosis and missed imported patients were not considered. In the process of calculating PRCC, in order to perform Latin hypercube sampling of the combined variables applied to the model, the distribution of each variable is assumed to follow a uniform distribution within 0.5–2 times that of the mean values used in the simulation. Table 3 shows the values of PRCC and p (2,000 trials of simulation performed).

It could be confirmed from the PRCC results that the number of secondary infection cases was most sensitive to the rate of transmission of infection of hospitalized patients against healthcare workers and the duration of confirmed diagnosis and isolation among hospitalized patients. Furthermore, the rate of transmission between healthcare workers did not significantly affect the number of secondary infected patients, as shown by the high p values of the PRCC results.

Table 3: PRCC of parameters and p-value

Parameter	PRCC	p-value
β_{CC}	0.3346	$<10^{-3}$
β_{HC}	0.1127	$<10^{-3}$
β_{HH}	0.0154	0.49
β_{JC}	0.2562	$<10^{-3}$
β_{JH}	0.9537	$<10^{-3}$
$1/\alpha_C$	0.3441	$<10^{-3}$
$1/\alpha_H$	0.0873	$<10^{-3}$
$1/\delta$	0.9615	$<10^{-3}$

References

[1] World Health Organization. Ebola virus disease 2018. Available from: https://www.who.int/news-room/fact-sheets/detail/ebola-virus-disease

[2] World Health Organization. Ebola virus disease situation report. Available from: https://www.who.int/csr/disease/ebola/situation-reports/archive/en/

[3] World Health Organization. Ebola data and statistics. 2016. Available from: http://apps.who.int/gho/data/view.ebola-sitrep.ebola-summary-latest?lang=en

[4] CNN. First diagnosed case of Ebola in the U.S. September 30, 2014. Retrieved October 27, 2014. Available from: https://edition.cnn.com/2014/09/30/health/ebola-us/index.html

[5] Onishi, Norimitsu. Liberian Officials Identify Ebola Victim in Texas as Thomas Eric Duncan. The New York Times. 2014. Available from: https://www.usatoday.com/story/news/nation/2014/10/02/liberia-ebola-patient-thomas-duncan-airport-screening/16591753/

[6] NBC News. New York Doctor Just Back From Africa Has Ebola. October 23, 2014. Retrieved October 23, 2014. Available from: https://www.nbcnews.com/storyline/ebola-virus-outbreak/new-york-doctor-just-back-africa-has-ebola-n232561

[7] Statistics Korea, Population estimates 2017-2067, 2019

[8] The Ministry of Health and Welfare, Statistical yearbook of health and welfare, 2019

[9] Diekmann O, Heesterbeek JA, Metz JA. On the definition and the computation of the basic reproduction ratio R0 in models for infectious diseases in heterogeneous populations. J Math Biol 1990; 28: 365–382.

[10] Gillespie DT. Exact stochastic simulation of coupled chemical reactions. J Phys Chem 1977;81:2340–2361.

[11] Marino S, Hogue IB, Ray CJ, Kirschner DE. A methodology for performing global uncertainty and sensitivity analysis in systems biology. J Theor Biol 2008;254(1):178–196. doi:10.1016/j.jtbi.2008.04.011

[12] Worldometers. population statistics. Available from: https://www.worldometers.info/world-population/sierra-leone-population/

[13] Government of Sierra Leone, Ministry of Health and Sanitation. Annual Health Sector Performance Report 2016. 2016, 25-27.
