# Supplementary material for: Ebola virus disease outbreak in Korea: use of a mathematical model and stochastic simulation to estimate risk
Source: Epidemiol Health. 2019 Nov 24;41:e2019048. doi: 10.4178/epih.e2019048 (PMC7005456; doi:10.4178/epih.e2019048)
Supplement: Supplementary file 3 [file epih-41-e2019048-suppl.docx]

**Ebola virus disease outbreak in Korea: use of a mathematical model and stochastic simulation to estimate risk**

Youngsuk Ko1, Seok-Min Lee2, Soyoung Kim1, Moran Ki3, Eunok Jung1

1*Department of Mathematics, Konkuk University, Seoul, Korea;* 2*Department of Liberal Arts, Hongik University College of Engineering, Seoul, Korea;* 3*Department of Cancer Control and Population Health, Graduate School of Cancer Science and Policy, National Cancer Center, Goyang, Korea*

Abstract

목적(Objectives): WHO에 의하면 2014년 서아프리카의 에볼라바이러스병(EVD) 대유행 이후 지속적으로 EVD 환자가 보고되고 있다. 본 연구에서는 수학적 모델링을 이용하여 EVD 감염자가 우리나라에 유입되었을 시 확산 규모를 다양한 조건에 따라 예측하고자 한다.

방법(Methods): 결정론적 수학적 모델(SEIJR or SEIJQR)에 기반한 서아프리카 EVD 확산 모델을 우리나라 상황에 맞게 변형하여 대한민국의 EVD 확산 모델을 구축하였고, 확률 시뮬레이션을 통해 국내에서 발생할 예상 환자수와 유행기간을 추정하였다. 또한 첫 번째 유입환자의 진단이 늦어지는 경우(Diagnosis delay)와 2차 감염자가 여러 명 발생할 때까지 EVD 인지를 못한 경우(Case missing)의 시나리오를 가상하여 EVD 유행기간, 최대 일일 발생 환자수, 총 발생 환자수를 예측하였다.

결과(Results): 각 시나리오별 2000번의 확률 시뮬레이션 시행 결과, 국내 첫 유입환자 의료기관 내원 즉시 중재정책을 적용한 경우 발생 가능한 환자 수는 중위수 2명, 최대 11명으로 예측되었다. 첫 국내 유입환자의 확진 날짜가 6일 늦어지는 경우는 중위수 7명, 최대 20명까지 환자가 발생할 수 있다. 첫 국내 유입환자를 확진하지 못하고 2명의 2차감염자가 발생할 때까지 정부의 중재정책이 시행되지 않는다면 중위수 15명, 최대 35명의 환자가 발생할 것으로 예측되었다.

결론(Conclusions): 국내에 신종 감염병이 유입되는 경우 확산 규모를 줄이기 위해서는 첫 국내 유입환자의 확진이 신속하고 철저하게 이루어지는 것이 가장 중요하다. 또한 2차 감염자를 놓치지 않도록 지역사회 감시체계와 진단체계를 강화하는 것이 필요하다.

중심단어(Key words)

에볼라바이러스병(Ebola virus disease), 수학적 모델(Mathematical model), 확률 시뮬레이션(Stochastic simulation), 중재 정책(Intervention strategy), 대한민국(Republic of Korea)

**List of English abbreviation**

EVD: Ebola Virus Disease

HCW: Health care workers

S: Susceptible

E: Exposed

I: Infectious

J: Hospitalized

Q: Isolation treated

R: Recovered

PHEIC: Public Health Emergency of International Concern

PRCC: partial rank correlation coefficient

**INTRODUCTION**

에볼라바이러스병(EVD: Ebola Virus Disease)은 에볼라바이러스에 의해 나타나는 전신성 질병으로 출혈을 동반하는 경우가 있어 출혈열(hemorrhagic fever)이라고도 불린다. EVD에 감염될 경우 약 8-10일(범위, 2-21일)의 잠복기 후에 갑자기 시작되는 발열, 허약, 두통, 근육통, 인후염 등의 증상이 나타나고(the dry phase), 병이 진행되면서 구토, 설사 등의 증상(the wet phase)을 보이게 된다. 발진, 신장과 간기능 저하, 일부환자에서는 내부와 외부 출혈이 나타나며 회복될 경우 발병 이후 10-12일 후 증상이 호전된다[1, 2, 3]. 감염 전파는 증상 시작과 함께 가능하며 보통 1미터 이내의 거리에서 체액이 전달되는 밀접접촉에 의하여 이루어지며 특히 환자가 사망한 상태에서 많은 바이러스 전파가 이루어진다[4, 5].

EVD 환자는 1976년 최초로 보고되었다. 이후 가장 큰 유행으로 2014-15년에 서아프리카(기니, 라이베리아, 시에라리온)에서 총 28,616명의 감염자와 11,310명의 사망자가 보고되었다[6]. EVD는 밀접접촉에 의하여 전파되는 특성과 높은 치사율로 인하여 대유행으로 발전되지 않는 경향을 가지고 있지만, 첫 유행이 3개국의 접경지인 교통중심지에서 시작되었고 사망자와 포옹을 하거나 키스를 하는 등의 서아프리카 장례문화로 인해 대규모로 확산되었으며, 또한 환자와의 밀접접촉이 상대적으로 많을 수밖에 없었던 의료진의 경우 높은 감염 위험에 대한 보고가 있었다[7-12]. 2014년 유행당시 서아프리카로 의료진을 파견했던 미국, 스페인, 영국에서도 사망자 및 감염자가 발생했다[8, 9, 10]. 미국에서는 서아프리카에서 EVD가 확진 되어 치료목적으로 귀국한 경우의 7건과 EVD 잠복기 상태로 귀국한 2건이 보고되었다. 잠복기 상태로 귀국한 사람 중 1명은 의료종사자가 아니었으며 해당 사건에서 미국 내에 추가적으로 2명의 의료진 감염이 발생했다[13, 14, 15]. 이후 EVD 유행이 사라지는 듯하였으나 2018년 5월부터 Democratic Republic of the Congo (DR Congo)에서 시작된 유행은 지속적으로 확대되어 2019년 7월 17일에는 WHO에서 다시 Public Health Emergency of International Concern (PHEIC)을 선포하였다.

감염병의 수학적 모델링은 실험적 관찰을 통한 귀납적 연구의 한계 극복의 과정이라는 점에서 국제적으로 중요한 역할을 하고 있다. EVD에 대해서도 많은 수학자들이 모델을 개발하였으며, 서아프리카 EVD 유행에 대해 장례문화를 고려한 수학적 모델도 소개되었다[16, 17].

본 연구에서는 수학적 모델링과 이를 기반으로 한 확률 시뮬레이션을 이용하여, EVD 잠복기 상태의 환자가 국내로 유입되었을 때 첫 국내 유입환자 확진 시기가 늦어지는 경우(diagnosis delay)와 2차 감염자가 여러 명 발생 때까지 EVD 유행을 인지 못한 경우(case missing)에 따른 예측환자 수를 계산하여 첫 환자 확진 시기와 조기 격리의 중요성에 대해 분석하고자 한다.

**MATERIALS AND METHODS**

데이터

모델의 모수 추정을 위하여 WHO의 2014년 서아프리카 시에라리온 EVD 유행 시 발생 환자 보고 데이터 중 최초 사망자 보고일인 2014년 5월 26일부터 마지막 보고가 기록된 2015년 5월 11일까지의 데이터를 사용하였다[6, 12]. 시에라리온 총 인구 데이터는 Worldometers의 2014년 인구 수를 이용하였으며 의료종사자 데이터는 한국의 의료종사자 데이터와 비슷한 기준을 적용하기 위하여 시에라리온 정부기관 데이터 CHO/CHA, Env/public health, nursing, midwife, other allied health, medical officer, nurse specialist 합계의 근삿값을 이용하였다[24, 25]. 대한민국의 지역사회 인구 및 의료종사자 수는 각각 통계청과 보건복지부 연감의 국민 10000명 당 의료종사자 수(의사, 간호사)를 이용하였다[18, 19].

수학적 모델

EVD 전파의 중요한 경로 중에 하나는 환자와의 접촉이 빈번한 의료진 감염과 감염된 의료진에 의한 전파로 알려져 있다. 따라서 본 연구에서는 의료종사자와 지역사회 환자의 감염 이질성을 고려하기 위하여 전체 인구를 의료종사자(healthcare worker; HCW)와 지역사회(community; C) 그룹으로 나누었다. **인구 집단은 의료종사자그룹과 지역사회그룹으로 나뉜 뒤 각각 감수성자 그룹(Susceptible; S), 잠복기 그룹(Exposed; E), 감염자 그룹(Infectious; I), 내원 환자 그룹(Hospitalized; J), 격리 치료자 그룹(Isolation treated; Q) 그리고 회복자(Recovered; R) 그룹으로 나누어진다. 확진 후 격리 치료 동안은 감염이 전파되지 않는다고 가정하였다. 격리 치료자 그룹과 회복자 그룹은 의료종사자 그룹과 지역사회 그룹간 행동패턴과 접촉율, 질병의 전파율 등이 동일하므로 의료-비의료 그룹을 나누지 않았다. Figure 1은 EVD 감염 전파의 흐름도를 나타낸 것이며 하늘색으로 병원 구역을 지역사회 구역과 구별이 되도록 표기하였다.**

**대한민국 EVD의 수리모델 구축을 위하여 2014년 시에라리온 데이터를 이용한 서아프리카 EVD 유행 모델(보충자료 참고)을 우리나라 의료상황에 맞게 변형하였다. 먼저, 서아프리카에서는 장례문화에 의한 감염이 중요한 경로였지만 국내 의료환경에는 시신에 의한 감염이 이루어지지 않는다 가정하였다. 또한, EVD 증상 환자는 모두 의료기관에 내원하여 격리된다고 가정하였다. EVD 증상 발현일부터 의료기간 내원일까지의 기간과 격리 기간 등은 2014년 미국 EVD 유입사례를 참고하였다.**

Figure 1 Flowchart of EVD model

EVD 바이러스에 한번도 걸린 적이 없는 감수성자 그룹은 감염자와 내원환자에 의해 EVD에 노출된다. 이 때 바이러스의 전파는 감염자와 내원환자가 지역사회 구성원인지 의료종사자인지에 따라 다르게 나타난다고 가정하였다. 각 항의 아래 첨자 H와 C는 각각 의료종사자 그룹과 지역사회 그룹을 나타낸다. 모델 상수 $\beta$는 감염 전파율을 의미하며, $\beta$의 아래 첨자는 전파 경로를 나타낸다. 예를 들어 $\beta_{CH}$는 지역사회의 감염자가 의료종사자에게 바이러스를 전파시키는 전파율을 나타낸다. 각 집단 별 상이한 감염전파율을 조합하여 $\lambda$로 표기하였다. 바이러스에 노출된 환자들은 일정기간이 지난후에 증상이 발현되어 전파력이 있는 감염환자로 발전하게 된다. 상수 $\kappa$는 EVD의 증상발현 진행률을 의미하고 $1/\kappa$은 EVD의 평균 잠복기간을 나타낸다. 증상이 발현된 환자는 일정기간 이후에 병원에 내원할 것이다. 감염환자의 내원율은 $\alpha$로 표기하였으며 $1/\alpha$는 증상 발현 이후 의료기관에 내원하기까지의 평균 기간이다. 내원환자는 EVD 확진 검사를 통해 EVD 확진 환자로 진행되고 EVD 확진 환자는 격리된다고 가정하였다. 내원환자가 확진 되어 격리되는 비율은 상수 $\delta$로 정의하고 $1/\delta$는 내원한 환자에 대한 확진 후 격리되기까지의 평균 기간을 나타낸다. 상수 $\gamma$는 격리 환자의 회복률을 나타내며 $1/\gamma$는 회복되기까지의 평균 격리기간을 의미한다. 격리가 된 이후에는 감수성 환자에게 전파시키지 않는다고 가정하였다. EVD 감염 전파의 수학적 모델은 다음과 같이 표현된다.

$$\frac{{dS}_{C}}{dt}=-\lambda_{C}S_{C}, \frac{{dS}_{H}}{dt}=-\lambda_{H}S_{H},$$

$$\frac{{dE}_{C}}{dt}=\lambda_{C}S_{C}-\kappa E_{C}, \frac{{dE}_{H}}{dt}=\lambda_{H}S_{H}-\kappa E_{H}$$

$$\frac{{dI}_{C}}{dt}=\kappa E_{C}-\alpha_{C}I_{C}, \frac{{dI}_{H}}{dt}=\kappa E_{H}-\alpha_{H}I_{H},$$

$$\frac{{dJ}_{C}}{dt}=\alpha_{C}I_{C}-\delta J_{C}, \frac{{dJ}_{H}}{dt}=\alpha_{H}I_{H}-\delta J_{H},$$

$$\frac{dQ}{dt}=\delta(J_{C}+J_{H})-\gamma Q,$$

$$\frac{dR}{dt}=\gamma Q,$$

$$\lambda_{C}=\frac{\beta_{CC}I_{C}+\beta_{HC}I_{H}+\beta_{JC}(J_{C}+J_{H})}{N},$$

$$\lambda_{H}=\frac{\beta_{CH}I_{C}+\beta_{HH}I_{H}+\beta_{JH}(J_{C}+J_{H})}{N},$$

$$N=S_{C}+E_{C}+I_{C}+J_{C}+S_{H}+E_{H}+I_{H}+J_{H}+Q+R.$$

한국형 EVD 감염 전파 모델의 모수 정의와 값은 Table 1과 같다. 감염전파율($\beta$)은 세계보건기구에 보고된 2014년 서아프리카 EVD 유행 당시 시에라리온 EVD 신환자수 데이터로부터 추정하였다. EVD 유행 데이터는 WHO 주별 누적 환자수 데이터와 모델에서의 해당 날짜의 누적 환자수를 비교, 최소제곱법(least square fitting method)을 사용하여 데이터와 모델에서 추정되는 신환자수 차이의 제곱을 최소화하는 감염전파율들을 추정하였다 [6]. 모수 추정에서 지역사회 그룹과 의료종사자 그룹 간의 전파율은 방향성을 고려하지 않았다. 즉, $\beta_{HC}=\beta_{CH}$이다. 시에라리온 데이터를 이용한 EVD 감염 전파율 추정의 구체적인 과정은 보충자료에 설명되어 있으며 시신에 의한 전파율을 제외한 모든 전파율은 서아프리카 모델과 동일하게 사용되었다. 모델에서 전체 감수성자 수($S_{C}(0)$+$S_{H}(0)$)는 51,709,000명이며 그 중 의료종사자수($S_{H}(0)$)는 558,970명이다[18, 19]. 최초 국내 유입 환자는 지역사회 그룹의 잠복기 환자 한 명으로 가정하였다($E_{C}(0)=1$).

EVD 감염 전파의 수학적 모델에서 Next-generation method를 이용하여 계산한 EVD의 기초감염재생산수($R_{0}$)는 다음과 같다 [20]. 여기서 $N_{C}$와 $N_{H}$는 각각 지역사회와 의료종사자 인구를 나타낸다.

$$R_{0}=\frac{A+\sqrt{A^{2}+4B}}{2},$$

$$A=\frac{p_{C}\beta_{CC}}{\alpha_{C}}+\frac{\beta_{HH}}{\alpha_{H}}p_{H}+\frac{\beta_{JC}}{\delta}p_{C}+\frac{\beta_{JH}}{\delta}p_{H},$$

$$B=p_{C}p_{H}\left( \frac{\beta_{HC}\beta_{JC}}{\alpha_{C}\delta}+\frac{\beta_{HC}\beta_{JH}}{\alpha_{H}\delta}+\frac{\beta_{HC}^{2}}{\alpha_{C}\alpha_{H}}-\frac{\beta_{CC}\beta_{HH}}{\alpha_{C}\alpha_{H}}-\frac{\beta_{CC}\beta_{JH}}{\alpha_{C}\delta}-\frac{\beta_{HH}\beta_{JC}}{\alpha_{H}\delta} \right),$$

$$p_{C}=\frac{N_{C}}{N}, p_{H}=\frac{N_{H}}{N}.$$

**Table 1. Model parameters to estimate outbreak size of Ebola Virus Disease**

| Symbol | Description | Value | Reference |
| --- | --- | --- | --- |
| $\beta_{CC}$ | Transmission rate between community members | $0.1352$ | Data-fitting |
| $\beta_{HC}, \beta_{CH}$ | Transmission rate between community and HCW | $0.811$ | Data-fitting |
| $\beta_{HH}$ | Transmission rate between HCW | $0.811$ | Data-fitting |
| $\beta_{JC}, \beta_{CJ}$ | Transmission rate between hospitalized and community | $0.0405$ | Data-fitting |
| $\beta_{JH}$ | Transmission rate between hospitalized and HCW | $45.5512$ | Data-fitting |
| $1/\kappa$ | Incubation period | 11 days | [1] |
| $1/\alpha_{C}$ | Period of symptom onset to hospitalization of community | 4 days | [13-15] |
| $1/\alpha_{H}$ | Period of symptom onset to hospitalization of HCW | 3 days | [13-15] |
| $1/\delta$ | Period of hospitalization to isolation | 2 days | [13-15] |
| $1/\gamma$ | Period of isolation to recovered | 14 days | [13-15] |

국내의 EVD 전파 확산 수학적 모델에 기반하여 개별 사건 단위로 진행되는 확률 모델 시뮬레이션 방법인 Gillespie 알고리즘을 이용하여 EVD 국내 유입 시 대응 시나리오에 따른 발생 환자수와 유행기간을 예측하였다 [21]. 본 연구에서 유행기간(outbreak duration)은 최초 환자 유입부터 마지막 환자의 치료 완료(recovered)까지의 기간으로 정의하였다. 개별 사건 단위로 시뮬레이션이 진행되는 Gillespie 알고리즘의 특성으로 인해 효율성 문제가 발생할 수도 있었지만 EVD 전파 상황에선 적은 수의 환자가 발생되므로 효율성 문제는 발생하지 않았다. Gillespie 알고리즘을 이용한 확률론적 시뮬레이션에서 사건 간의 시간간격이 감염이나 입원, 격리 및 회복에 대한 propensity들의 합에 반비례한다. 각 사건들의 propensity는 Table 2에 표기되어 있다.

**Table 2. Possible transitions and propensities in the model**

| Transitions | Propensity |
| --- | --- |
| $S_{C}\to S_{C}-1$, $E_{C}\to E_{C}+1$ | $\frac{\beta_{CC}I_{C}+\beta_{HC}I_{H}+\beta_{JC}(J_{C}+J_{H})}{N}S_{C}$ |
| $E_{C}\to E_{C}-1$, $I_{C}\to I_{C}+1$ | $\kappa E_{C}$ |
| $I_{C}\to I_{C}-1$, $J_{C}\to J_{C}+1$ | $\alpha_{C}I_{C}$ |
| $J_{C}\to J_{C}-1$, $Q\to Q+1$ | $\delta J_{C}$ |
| $S_{H}\to S_{H}-1$, $E_{H}\to E_{H}+1$ | $\frac{\beta_{CH}I_{C}+\beta_{HH}I_{H}+\beta_{JH}(J_{C}+J_{H})}{N}S_{H}$ |
| $E_{H}\to E_{H}-1$, $I_{H}\to I_{H}+1$ | $\kappa E_{H}$ |
| $I_{H}\to I_{H}-1$, $J_{H}\to J_{H}+1$ | $\alpha_{H}I_{H}$ |
| $J_{H}\to J_{H}-1$, $Q\to Q+1$ | $\delta J_{H}$ |
| $Q\to Q-1$, $R\to R+1$ | $\gamma Q$ |

민감도 분석

편순위상관계수방법(partial rank correlation coefficient, PRCC)를 이용하여 수리모델의 모수 변화가 모델 결과에 미치는 영향을 분석하였다 [22]. 본 연구에서는 감염전파율($\beta_{CC}$,$\beta_{HC}$,$\beta_{HH}$,$\beta_{JC}$)과 증상 발현 후 내원까지의 기간($1/\alpha_{C}$, $1/\alpha_{H}$), 그리고 내원 환자의 확진 및 격리까지의 기간($1/\delta$)을 변수로 설정한 뒤 전파기간동안 발생하는 2차 감염자 수를 해당 변수에 대한 함수로 설정하였다. 민감도 분석에서 진단 지연 및 유입 미인지 상황은 고려하지 않았다. PRCC 계산 과정에서 모델 적용 변수들의 조합에 대한 라틴 하이퍼큐브 샘플링**(Latin hypercube sampling)**을 위해 각 변수들의 분포는 시뮬레이션에 사용된 값을 평균으로 0.5에서 2배 사이 값 사이에서 **균등분포(uniform distribution)를** 따른다 가정하였다. Table 3는 편상관계수값과 p 값을 나타낸다(2000회 시행).

**Table 3. PRCC of parameters and p-value**

| Parameter | PRCC | p-value |
| --- | --- | --- |
| $\beta_{CC}$ | 0.3346 | <${10}^{-3}$ |
| $\beta_{HC}$ | 0.1127 | <${10}^{-3}$ |
| $\beta_{HH}$ | 0.0154 | 0.49 |
| $\beta_{JC}$ | 0.2562 | <${10}^{-3}$ |
| $\beta_{JH}$ | 0.9537 | <${10}^{-3}$ |
| $1/\alpha_{C}$ | 0.3441 | <${10}^{-3}$ |
| $1/\alpha_{H}$ | 0.0873 | <${10}^{-3}$ |
| $1/\delta$ | 0.9615 | <${10}^{-3}$ |

PRCC결과를 통해2차 감염자 수는 내원 환자의 의료종사자에 대한 감염전파율과 내원환자의 확진 및 격리기간에 가장 민감하다는 것을 확인할 수 있었다. 또한 의료종사자간의 전파율은 PRCC의 높은 p 값을 통해 2차 감염자 수에 유의미하지 않았다는 점을 알 수 있었다.

국내 유입 확산 시나리오

감수성 환자가 전염성이 있는 환자와의 접촉을 통해 바이러스에 노출된 이후, 잠복기($E$)를 거쳐 증상이 나타나면($I$) 병원에 내원($J$)하고, 병원에서 EVD 확진이 된 이후에 격리($Q$)될 것이다. 증상 발현 이후 격리되기 전까지의 환자들($I$, $J$)로부터 EVD가 전파될 수 있다. EVD 환자가 최초로 확진된 후 정부의 EVD 중재 정책이 시행된다고 가정하였다. 중재 정책이 시행되면 증상 발현부터 내원까지의 기간이 지역사회에서는 4일에서 2일, 병원내에서는 3일에서 2일로 감소하며, 내원 후 확진 및 격리까지의 기간은 2일에서 1일로 감소한다고 가정하였다. 또한 사람들이 사회적으로 밀접접촉을 자제하여 감염전파율($\beta_{CC}$,$\beta_{HC}$,$\beta_{HH}$,$\beta_{JC}$)이 20% 감소하며, 병원 내에서도 환자에 의한 의료종사자로의 감염전파율($\beta_{JH}$)은 60% 감소할 것이라 가정하였다(Table 4).

**Table 4. Model parameters before and after intervention for Ebola Virus Disease outbreak**

| Symbol | Pre-behavior change | Post-behavior change |
| --- | --- | --- |
| $\beta_{CC}$ | $0.1352$ | $0.1082$ |
| $\beta_{HC}$ | $0.0811$ | $0.0649$ |
| $\beta_{HH}$ | $0.0811$ | $0.0649$ |
| $\beta_{JC}$ | $0.0405$ | $0.0324$ |
| $\beta_{JH}$ | $45.5512$ | $18.2205$ |
| $1/\alpha_{C}$ | 4 days | 2 days |
| $1/\alpha_{H}$ | 3 days | 2 days |
| $1/\delta$ | 2 days | 1 day |

EVD 국내 유입 대응 시나리오는 다음과 같이 Best 시나리오 및 감염 확산을 고려한 추가적인 두 상황으로 구성하였다.

Best 시나리오($\mathbb{S}I$) : 국내 유입환자 의료기관 내원 즉시 확진 및 격리 진행 (미확진 환자 0명, 진단 지연 0일)

Diagnosis delay 시나리오($\mathbb{S}\mathrm{II}$) : 국내 유입환자 내원 후 3일 또는 6일까지 진단 지연

Case missing 시나리오($\mathbb{S}\mathrm{III}$) : 2차 감염자가 1명 또는 2명 내원할 때까지 EVD 유입 미인지

**RESULT**S

지역사회 그룹 인원 한 명이 잠복기 상태로 국내 유입된 후 120일간의 EVD 감염 전파 양상을 분석하기 위해 확률 시뮬레이션 방법인 Gillespie 알고리즘을 적용, 2000번의 시행을 하여 결과를 도출하였다. 시뮬레이션 결과 분석 중 총 환자수(I)는 수학적 모델에서 지역사회 감염자($I_{C}$)와 병원내감염자($I_{H}$)의 합이다.

Figure 2는 Best 시나리오($\mathbb{S}I$)에 대한 Gillespie 알고리즘 2000번의 시행 중 무작위로 추출된 5개 시행의 환자수와 전체 시행 평균 환자수, 그리고 95% 신뢰구간 upper bound 환자수의 변화추이를 각각 회색 곡선들과 빨간색 실선, 그리고 빨간색 점선으로 표시하였다. 본 연구에서 95% 신뢰구간 upper bound는 전체 2000번 시행의 97.5%에 해당하는 percentile로 정의하였다. 시뮬레이션 결과 첫 환자 유입 25일 후 95% 신뢰구간 내에서 최대 3.5명 정도의 총 환자가 발생할 수 있고, 전체 시행에서 평균적으로 1명 이내의 환자가 발생함을 보였다.

Figure 3은 각 시나리오에 대한 예측환자 수와 마지막 환자가 회복되는 시점인 유행기간의 시뮬레이션 결과 분포를 상자 그림으로 보여준다. 상자 그림에서 표시하는 각 경우의 중위수와 신뢰구간 최대 최소의 수치는 Table 5에 제시되어 있다. Table 4에서는 EVD 대응 시나리오별 예측환자 수(지역사회(Community)와 의료종사자(HCW) 그룹별 총 예측환자 수, 중위수와 신뢰구간 최대와 최소), 일일 최대 발생자수(maximum number of new cases per day), 유행 기간, 그리고 전체 시행 중 총 예측환자수가 10명, 20명, 30명 이상 발생하는 시행의 비율로 계산된 확률(Probability of estimated total case) 및 최초 확진에 의해 중재 정책 시행 이전 재생산지수를 보여준다.

Best 시나리오($\mathbb{S}I)$에서는 총 환자수 예측값의 중위수가 2명 그리고 최대 11명으로 계산되었고, 10명 이상의 환자가 발생할 확률은 4.1%이었으며, 유행기간의 중위수는 약 44일이었다. 이는 흥미롭게도 미국의 EVD 발생 사례들과 유사한 결과이다[13-15].

국내 유입환자의 진단 지연을 가정한 Diagnosis delay 시나리오($\mathbb{S}II)$의 경우, 국내 유입환자의 확진이 3일 지연될 경우 지역사회 감염자가 2명과 병원내 감염자가 3명, 총 5명의 환자가 발생할 것으로 예측된다. 일일 최대 환자는 국내유입 첫 환자 발생 23일째에 2명이 발생할 것으로 예측되고, 유행기간은 약 2달(58일) 정도 지속될 것으로 예측된다. 확진이 6일 지연될 경우에는 총 7명의 환자가 발생할 것으로 예측되며 유행기간은 69일정도 지속될 것으로 예측된다.

다음은 2차감염자가 추가로 발생할 때까지 정부가 EVD유입을 인지 못한다고 가정한 Case missing 시나리오($\mathbb{S}III)$을 고려하였다. 2차 감염자가 1명 발생까지 EVD 유입을 인지하지 못 할 경우, 감염자의 중위수는 지역사회 3명과 병원내 감염 5명으로 총 8명의 환자가 발생할 것이라고 예측된다. 국내 유입 첫 환자 발생후 24일째에 일일 최대 3명의 환자가 발생될 것으로 예상되며, 유행기간은 약 77일정도 지속될 것이라고 예측되었다. 1명의 2차감염자가 발생할 때까지 EVD 유입을 인지하지 못한 경우는 국내 유입환자의 진단이 약 8일 지연되는 상황과 같았다. 이 경우에는 약 88일의 유행기간 동안 감염자의 중위수는 지역사회가 5명 병원내 감염자가 10명이 발생하고, 국내유입 첫 환자 발생24일 후에 하루 5명의 환자가 발생할 것이라고 예측되었다. 2차 감염자가 2명 발생할 때까지 EVD유행을 인지하지 못하는 경우 10명이상의 환자가 발생할 확률이 75.7%로 2차감염자가 1명 발생할 때까지 EVD 유행을 인지 못하는 경우(32.8%)보다 2배 이상 높게 나타났다.

감염재생산수($R_{0}$)는 $\mathbb{S}I$ 기준으로 중재 정책 시작 전은 2.42, 중재 정책 시작 이후로는 0.76으로 감소하였다. 진단이 지연되는 시나리오에선($\mathbb{S}$II), 3일(6일)의 진단 지연으로 인해 최초 유입 환자는 기존의 감염재생산수 2.42보다 높은 4.05(6.05) 값을 보였다. 또한 만약 확진 및 격리까지의 시간을 기존의 평균 2일에서 6시간으로 단축시킬 경우, 중재 정책이 적용되지 않은 상황에서도 감염재생산수는 0.99로 1보다 작음을 확인하였다.

**Table 5. Estimated number of case and duration of outbreak of Ebola Virus Diseases by scenario**

|  | $\mathbb{S}I$ Best | $\mathbb{S}\mathrm{II}$ Diagnosis delay (days) | | $\mathbb{S}\mathrm{III}$ Case missing (n) | |
| --- | --- | --- | --- | --- | --- |
|  |  | 3 | 6 | 1 | 2 |
| Estimated number of case (median, (min-max)) |  |  |  |  |  |
| Total | 2 (1-11) | 5 (1-14) | 7 (1-20) | 8 (1-24) | 15 (1-35) |
| Community | 1 (1-6) | 2 (1-6) | 3 (1-8) | 3 (1-9) | 5 (1-13) |
| Hospital | 1 (0-5) | 3 (0-8) | 4 (0-12) | 5 (0-15) | 10 (0-22) |
| Maximum number of existing cases (max) | 1 (3) | 2 (4) | 2 (6) | 3 (7) | 5 (10) |
| Outbreak duration (days) (median, (min-max)) | 44 (3-121) | 58 (5-139) | 69 (11-152) | 77 (7-156) | 88 (27-164) |
| Probability of estimated total case (%) |  |  |  |  |  |
| 30 or more | 0 | 0 | 0.3 | 0 | 3.2 |
| 20 or more | 0.3 | 1.1 | 3.0 | 1.6 | 20.3 |
| 10 or more | 4.1 | 10.5 | 21.2 | 32.8 | 75.7 |
| Reproductive number  (Pre-behavior change) | 2.42 | 4.05 | 6.05 | 7.74 | 10.49 |

Figure 2 Estimated number of existing cases after the first patient of Ebola Virus Disease entered in Korea using model simulations. Grey curves are random sampled 5 simulations. Red curve indicates mean number of existing cases and dashed red curve indicates upper limit within 95% confidence interval.

**Figure 3. Boxplot results by scenario: SI is Best, SII is Diagnosis delay, and SIII is Case missing scenario. Number in parentheses of SII and SIII indicates diagnosis delay day and case missing number, respectively. On each box, red horizontal line indicates the median and bottom and top of each box indicates 25th and 75th percentiles, respectively. Whiskers reaches to the extreme points within confidence interval. Red crosses are outliers of the stochastic simulation.**

**DISCUSSION**

국내 EVD 유입 첫 환자의 진단 지연과 2차 감염자 발생은 EVD 환자수 및 유행기간 증가를 야기했다. 첫 국내 유입환자가 의료기관에 내원 후 3일동안 확진 및 격리가 진행되지 않는다고 가정한 경우(Diagnosis delay, $\mathbb{S}$II: 3 days) 첫 국내 유입환자 조기 확진 격리가 이루어지는 경우(Best, $\mathbb{S}$I)에 비해 지역사회 2차 감염자 1명, 의료종사자 2차 감염자 2명이 추가로 발생할 것이라고 예측되었다. 총 유행기간은 14일 증가할 것으로 예측되고 10명 이상의 환자가 발생할 확률은 10.5%로 국내 유입환자 조기 확진 격리시보다 6.4% 포인트 높게 나타났다. 첫 국내 유입환자의 진단지연이 6일이라고 가정한 시나리오(Diagnosis delay, $\mathbb{S}$II: 6 days)에서는 첫 유입환자 조기 확진 격리 시나리오보다 5명의 2차 감염자(지역사회 2명, 의료종사자 3명)가 추가로 발생할 것으로 예측되었으며 유행기간은 25일 증가할 것으로 보인다. 10명의 이상의 환자가 발생할 확률은 21.2%로 진단지연이 3일이 때보다 2배 이상 높게 예측되었다.

2차 감염자가 추가로 발생할 때까지 정부가 EVD 유입을 인지하지 못한다고 가정한 시나리오(Case missing, $\mathbb{S}$III)에서 2차 감염자 1명이 내원할 때까지 EVD 국내 유입을 인지하지 못한 경우, 첫 번째 유입환자가 조기 확진 격리되는 시나리오(Best, $\mathbb{S}$I)에 비해 2명의 지역사회 2차 감염자, 4명의 의료종사가 감염이 추가로 발생할 것이라고 예측되었다. EVD 유행은 33일 증가한 77일 지속될 것으로 보인다. 10명 이상의 환자가 발생할 확률은 32.8%로 첫 유입환자 조기 격리 시나리오보다 8배 높은 확률을 보였으며 20명 이상의 환자가 발생할 확률도 1.6%를 나타냈다. 2명의 2차 감염자가 의료기관에 내원할 때까지 EVD 유입인지를 못할 경우 13명의 2차 감염자가 추가로 발생하여 총 15명의 환자가 발생할 것이라고 예측되었다. 유행은 첫 유입환자 조기 격리 시나리오 보다 2배 증가하여 88일정도 지속될 것으로 보인다. 2차 감염자 1명이 발생할 때까지 EVD 유행을 인지하지 못한 경우와 2명 EVD 미인지인 경우의 유행기간에서는 큰 차이가 없었지만, 예측 총 환자수와 10명 이상 환자가 발생할 확률은 2배 이상, 그리고 20명 이상은 12.68배 정도로 2차 감염자가 2명 발생할 때까지 EVD 미인지인 경우에 유행의 규모가 훨씬 커질 수 있다는 결과를 볼 수 있었다. 특히 국내 첫 유입 환자의 조기 확진과 격리를 하는 Best 시나리오($\mathbb{S}$I)와 비교한다면 총 환자수는 2명에서 15명으로 증가하고, 예측 총 환자수가 10% 이상 발생할 확률도 4.1%에서 75.7%로 1차 감염자 미확진으로 인한 2차 감염자 발생시 EVD 집단 발생이 일어날 수 있음을 시뮬레이션 결과가 보여주었다.

시뮬레이션에서 Case missing과 Diagnosis delay 시나리오의 Best 시나리오 대비 총 환자 수 증가 대부분은 의료종사자 그룹에서 발생하였다. EVD 전파의 특성상 의료종사자의 감염위험이 지역사회 감수성자의 감염위험보다 높게 나타나기 때문에 유행기간이 증가할수록 병원 내 EVD 미인지 내원환자에 의한 의료종사자의 감염위험이 증가했다고 판단된다. 또한 EVD 환자 유입을 인지함으로써 중재 정책 시행이 되기 전일지라도 최초 유입 환자의 증상 발현 이후 내원한 상황에서 빠른 진단 및 격리(6시간)를 통하여 감염재생산수를 1보다 작게 만들 수 있음을 확인하였다.

현재 우리 나라의 대형병원들은 병원당 최소 3명 이상의 환자를 수용할 수 있는 격리병실을 보유하고 있다 [23]. 첫 번째 환자를 지연 없이 확진 및 격리를 하는 시나리오(Best, $\mathbb{S}$I)에선 중위수로 2명의 환자가 발생할 것으로 예측되므로 중위수 기준에선 현재 우리 나라의 대형병원들은 충분한 음압격리병실을 보유하고 있다고 볼 수 있다. 하지만 신뢰구간에서 최대 11명까지 환자가 발생할 것으로 예측되고 수용 가능 인원보다 환자가 더 많이 발생할 경우, 타 대형병원으로의 이송 과정에서 추가적인 확산 및 의료진의 감염의 위험의 가능성이 있다. 또한 진단 지연과 진단 누락 시나리오(Case missing($\mathbb{S}$II), Diagnosis delay($\mathbb{S}$III)) 경우는 중위수 기준으로도 최소 5명의 환자가 발생할 것으로 예측되므로 한 병원에서 수용할 수 있는 인원을 초과한다고 볼 수 있다. 따라서 진단 지연과 진단 누락상황에 대한 대비가 필요하다고 볼 수 있다.

**CONCLUSION**

EVD는 밀접접촉 혹은 사망자의 체액 노출에 의하여 전파하는 특성이 있으므로, 사망자와의 접촉이 적은 대한민국에서는 환자가 유입되더라도 큰 유행으로 발전할 가능성은 적다. 하지만 환자들의 높은 사망률로 인하여 유행까지 발전하지 않더라도 관심을 가져야 하는 질병이고 의료종사자들의 높은 감염 노출 위험을 대비할 필요가 있다.

본 연구는 국내에서의 EVD 질병 전파 양상을 수학적 모델로 구축하여, EVD 감염 사실을 모르는 EVD 잠복기인 환자가 국내에 유입된 상황을 가정하고 미국과 유사한 중재정책을 고려하여 환자의 발생 규모와 유행기간을 예측하는 시뮬레이션을 수행하였다. 또한 Case missing 및 Diagnosis delay의 시나리오 하에서는 얼마나 환자의 규모가 얼마나 커지는지 혹은 유행기간이 얼마나 길어질 수 있는지 시뮬레이션을 수행하여 비교 및 분석을 하였다. 결과적으로, 미국과 유사한 EVD 경계 상황을 고려할 경우(Best 시나리오) 최초 유입 환자와 한 명의 의료종사자가 감염될 것으로 기대되지만, 첫 국내 유입환자의 진단이 6일 지연되는 경우 총 예측환자는 7명, 최대 20명까지 유행이 발생할 수 있음을 관찰하였다. 2차 감염자가 2명이 발생할 때까지 정부가 EVD 유행을 인지하지 못할 경우 총 환자가 중위수로 15명, 신뢰구간에서 최대 35명까지 발생할 수 있음을 관찰하였다.

EVD는 국내에 유입된 사례가 없었기 때문에 서아프리카의 시에라리온의 EVD 유행 데이터를 이용하여 모델의 모수들을 추정하였다. 우리나라에서의 감염 전파율은 사회적 접촉패턴이 다른 시에라리온과 같을 수 없지만 2015년 MERS 유행과 같이 감염원 국가보다도 감염 전파율이 높을 수도 있다는 점에서 보듯 반드시 우리 나라에선 감염 전파율이 낮을 것이라고 확신할 수는 없다. 모수의 불확실성을 부분적으로 극복하기 위하여 민감도 분석을 했으며, 시뮬레이션 결과를 통하여 내원환자의 빠른 격리와 병원내 감염 방지의 중요성을 확인할 수 있었다.

수리모델링과 시뮬레이션 결과에서, 해외에서 유입되는 감염병의 경우, 첫번째 감염환자가 최대한 빨리 확진 되어 바로 격리 치료 및 접촉자 추적 등 중재정책을 신속하게 실시하는 것이 매우 중요함을 알 수 있었다. 본 연구에서는 국내 전파에 따른 추가적인 환자가 발생시 진단의 어려움이나 접촉자 추적의 어려움은 가정하지 않았다. 국외 출입기록이 없는 2차 감염자가 EVD 유사증상으로 의료기관에 내원하였을 때는 EVD 확진이 더욱 늦어질 가능성이 있고 3차 감염자가 발생할 가능성이 높다. 따라서 신속한 환자 발견 및 확진을 위하여 해외 유입 감염병에 대한 모니터링 및 환자 추적, 내원 환자의 해외 체류 여부 조사 등의 대응체제 구축의 필요성도 강조할 수 있다.

최근 EVD 백신이 개발되어 2018-2019년 Congo EVD 대유행시 백신 접종이 실시되었다. 최근 EVD 백신이 개발되어 2018-2019년 Congo EVD 대유행시 백신 접종이 실시되었다. 본 연구에서는 대한민국 의료상황을 고려하여 최초 EVD 환자 유입시 지역사회와 의료종사자의 2차 감염자 발생에 대한 연구에 초점을 맞추었기 때문에 백신 접종을 고려하지 않았다. 추후 연구에서는 의료기관으로의 EVD 환자 유입에 대비한 의료종사자의 백신접종을 고려할 필요가 있다.

**REFERENCES**

1. World Health Organization. Ebola virus disease 2018. Available from: https://www.who.int/news-room/fact-sheets/detail/ebola-virus-disease
2. Kaner J, Schaak S. Understanding Ebola: the 2014 Epidemic. Globalization and Health 2016. 12:53.
3. Vetter P, Fischer WA 2nd, Schibler M, Jacobs M, Bausch DG, Kaiser L. Ebola Virus Shedding and Transmission: Review of Current Evidence. J Infect Dis 2016; 214(suppl 3): S177–S184. doi:10.1093/infdis/jiw254
4. Judson, Seth & Prescott, Joseph & Munster, Vincent. Understanding Ebola Virus Transmission. Viruses. 2015;7. 511-521. 10.3390/v7020511.
5. Francesconi P et al. Ebola hemorrhagic fever transmission and risk factors of contacts, Uganda. Emerging Infectious Diseases. 2003;9:1430–1437.
6. World Health Organization. Ebola virus disease situation report. Available from: https://www.who.int/csr/disease/ebola/situation-reports/archive/en/
7. Amanda Tiffany, Benjamin D. Dalziel, Hilary Kagume Njenge, Ginger Johnson, Roselyn Nugba Ballah, Daniel James, et al. Estimating the number of secondary Ebola cases resulting from an unsafe burial and risk factors for transmission during the West Africa Ebola epidemic. PLOS Neglected Tropical Diseases. 2017;11(6): e0005491. https://doi.org/10.1371/journal.pntd.0005491.
8. Centers for Disease Control and Prevention. 2014-2016 Ebola Outbreak in West Africa. Available from: https://www.cdc.gov/vhf/ebola/history/2014-2016-outbreak/index.html
9. Centers for Disease Control and Prevention. Overview, Control Strategies, and Lessons Learned in the CDC Response to the 2014–2016 Ebola Epidemic. Available from: https://www.cdc.gov/mmwr/volumes/65/su/su6503a2.htm
10. Gatherer D. The 2014 Ebola virus disease outbreak in West Africa. Journal of General Virology. 2014;95: 1619-1624, doi: 10.1099/vir.0.067199-0.
11. Health worker Ebola infections in Guinea, Liberia and Sierra Leone. World Health Organization; 21 May 2015.
12. World Health Organization. Ebola data and statistics. 2016. Available from: http://apps.who.int/gho/data/view.ebola-sitrep.ebola-summary-latest?lang=en
13. CNN. First diagnosed case of Ebola in the U.S. September 30, 2014. Retrieved October 27, 2014. Available from: https://edition.cnn.com/2014/09/30/health/ebola-us/index.html
14. Onishi, Norimitsu. Liberian Officials Identify Ebola Victim in Texas as Thomas Eric Duncan. The New York Times. 2014. Available from: https://www.usatoday.com/story/news/nation/2014/10/02/liberia-ebola-patient-thomas-duncan-airport-screening/16591753/
15. NBC News. New York Doctor Just Back From Africa Has Ebola. October 23, 2014. Retrieved October 23, 2014. Available from: https://www.nbcnews.com/storyline/ebola-virus-outbreak/new-york-doctor-just-back-africa-has-ebola-n232561
16. Legrand J, Grais RF, Boelle PY, Valleron AJ, Flahault A. Understanding the dynamics of Ebola epidemics. Epidemiol Infect. 2007;135(4):610–621. doi:10.1017/S0950268806007217.
17. Folashade B Agusto, Miranda I Teboh-Ewungkem, Abba B Gumel. Mathematical assessment of the effect of traditional beliefs and customs on the transmission dynamics of the 2014 Ebola outbreaks. BMC Medicine 2015;13:96 https://doi.org/10.1186/s12916-015-0318-3.
18. Statistics Korea, Population estimates 2017-2067, 2019
19. The Ministry of Health and Welfare, Statistical yearbook of health and welfare, 2019
20. Diekmann, J.A.P. Heesterbeek, and J.A.J. Metz. On the definition and the computation of the basic reproduction ratio R0 in models for infectious diseases in heterogeneous populations. J. Math. Biol. 1990; 365–382.
21. Danlel T. Gillespie. Exact Stochastic Simulation of Coupled Chemical Reactions. The J. Phys. Chem. 1977;2340–2361.
22. Marino S, Hogue IB, Ray CJ, Kirschner DE. A methodology for performing global uncertainty and sensitivity analysis in systems biology. J Theor Biol. 2008;254(1):178–196. doi:10.1016/j.jtbi.2008.04.011
23. Korea Centers for Disease Control and Prevention, Guidelines for the Operation and Management of State-designated Inpatient Care Hospitals, 2017
24. Worldometers. population statistics. Available from: <https://www.worldometers.info/world-population/sierra-leone-population/>
25. Government of Sierra Leone, Ministry of Health and Sanitation. Annual Health Sector Performance Report 2016., 2016, 25-27.
